# High‐Density Nanopore Confined Vortical Dipoles and Magnetic Domains on Hierarchical Macro/Meso/Micro/Nano Porous Ultra‐Light Graphited Carbon for Adsorbing Electromagnetic Wave

**DOI:** 10.1002/advs.202303217

**Published:** 2023-08-01

**Authors:** Wenhuan Huang, Xingxing Zhang, Jiamin Chen, Qiang Qiu, Yifan Kang, Ke Pei, Shouwei Zuo, Jincang Zhang, Renchao Che

**Affiliations:** ^1^ Key Laboratory of Chemical Additives for China National Light Industry College of Chemistry and Chemical Engineering Shaanxi University of Science and Technology Xi'an 710021 China; ^2^ Laboratory of Advanced Materials Shanghai Key Lab of Molecular Catalysis and Innovative Materials Academy for Engineering & Technology Fudan University Shanghai 200438 P. R. China; ^3^ Zhejiang Laboratory Hangzhou 311100 P. R. China

**Keywords:** electromagnetic wave absorber, electron holography, energetic metal‐organic framework, magnetic domain, vortical dipole

## Abstract

Atomic‐level structural editing is a promising way for facile synthesis and accurately constructing dielectric/magnetic synergistic attenuated hetero‐units in electromagnetic wave absorbers (EWAs), but it is hard to realize. Herein, utilizing the rapid explosive volume expansion of the CoFe‐bimetallic energetic metallic triazole framework (CoFe@E‐MTF) during the heat treatment, the effective absorption bandwidth and the maximum absorption intensity of a series of atomic CoFe‐inserted hierarchical porous carbon (CoFe@HPC) EWAs can be modified under the diverse synthetic temperature. Under the filler loading of 15 wt%, the fully covered X and Ku bands at 3 and 2.5 mm for CoFe@HPC800 and the superb minimum reflection loss (*RL*_min_) of −53.15 dB and specific reflection loss (*SRL*) of −101.24 dB mg^−1^ mm^−1^ for CoFe@HPC1000 are achieved. More importantly, the single‐atomic chemical bonding among Co─Fe on the nanopores is captured by extended X‐ray absorption fine structure, which reveals the formation mechanism of nanopore‐confined vortical dipoles and magnetic domains. This work heralds the infinite possibilities of atomic editing EWA in the future.

## Graphical abstract

Four CoFe‐inserted hierarchical porous carbon captured under different temperatures revealed the evolution of Co/Fe from clusters to single atom states through XANES and EXAFS. The LTEM and hologram in situ monitor the high‐density nano‐vortex dipoles and magnetic domains on 3D network. The bifunctional regulation of fully covered X/Ku bands and superb specific reflection loss of −101.24 dB mg^−1^ mm^−1^ are achieved.

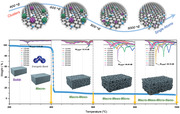

## 1 Introduction

Optimizing dielectric/magnetic synergetic attenuation ability with the balance of highly matched impedance is an effective approach for obtaining high electromagnetic wave (EW) absorbing performance.^[^ ^1^ ^]^ Recently, scientists have been dedicated to constructing highly dispersed magnetic units and chemical heterojunctions into highly porous dielectric materials, especially carbon‐based materials, which have promoted the blowout generation of a variety of excellent EW absorbers (EWAs).^[^ ^2^ ^]^ Among various magnetic metal‐based organic compounds or complexes, crystalline metal‐organic frameworks (MOFs) as precursors possess considerable merits of highly ordered and precise molecular structures.^[^ ^3^ ^]^ Their adjustable coordination structures and stable physicochemical properties contributed to the post‐synthesis of unique porous nanostructures, such as hollow,^[^ ^4^ ^]^ core–shell,^[^ ^5^ ^]^ yolk‐shell,^[^ ^6^ ^]^ multi‐layers,^[^ ^4^, ^7^ ^]^ and hierarchical pores,^[^ ^8^ ^]^ which have shown great performance on modifying the dipolar and interfacial polarization and impedance matching.

It is well‐known that the complex post‐synthesis ways have been applied to MOF precursors for obtaining these special micro‐nano structures such as multi‐step hydrothermal synthesis,^[^ ^9^ ^]^ chemical etching,^[^ ^3^, ^10^ ^]^ surfactant foaming,^[^ ^11^ ^]^ and electrochemical reactions.^[^ ^12^ ^]^ The complex technical processes, low metal utilization rates, and the resulting waste liquids have greatly hindered the large‐scale production and commercial applications of these materials. Hence, developing simple and gentle methods to obtain highly porous EWAs, especially hierarchical porous architectures with low density, has attracted the great attention of scientists. For instance, polymer‐bubbling,^[^ ^13^ ^]^ supercritical CO_2_ foaming,^[^ ^14^ ^]^ freeze‐drying,^[^ ^15^ ^]^ and alkane‐blowing agents,^[^ ^16^ ^]^ have delivered a variety of excellent hierarchical porous EWAs. However, such in situ assembly processes were unfavorable for the precise construction of highly dispersed magnetic metallic hetero‐units into the structure owing to the aggregation of metals at high temperatures.

Atomic‐level structure editing from bottom to up may satisfy both the demands of facile synthesis and accurate building dielectric/magnetic synergetic hetero‐units in hierarchical porous architecture, which is very hard to realize. Herein, we designed and synthesized a CoFe‐bimetallic energetic metallic triazole framework (CoFe@E‐MTF) from 1H‐1,2,3‐triazole under room‐temperature reaction. Utilizing the rapid volume expansion of the CoFe@E‐MTF caused by the decomposition of high‐energetic N_3_‐bonds, we controlled the calcinated temperatures (≈400–1000 °C) and successfully captured four atomic CoFe‐inserted hierarchical porous carbon (CoFe@HPC*x*, *x* = 400, 600, 800, 1000 °C) in diverse states. In such a series of EWAs, the atomic chemical bonding and existence status of Co and Fe in materials were deeply analyzed by X‐ray absorption near‐edge structure (XANES) and extended X‐ray absorption fine structure (EXAFS) spectra, revealing the structural evolution of Co and Fe from the cluster to single atom state on the carbon matrix. The Lorentz transmission electron microscopy (LTEM) and hologram imaging in situ monitor the formation and distribution of high‐density nano‐vortex dipoles and magnetic domains on a 3D conductive network, which is determined by the atomic Co─Fe coupling diploes. The polarization and magnetic exchange enhanced the EW attenuation ability with the modified impedance matching. As a result, under the filler loading of 15 wt%, the bifunctional regulation of fully covered X and Ku bands at 3 and 2.5 mm for CoFe@HPC800 and the superb minimum reflection loss value (*RL*_min_)of −53.15 dB and specific reflection loss ( *SRL)* of −101.24 dB mg^−1^ mm^−1^ for CoFe@HPC1000 were achieved.

## 2 Results and Discussion

### 2.1 Energetic Building Block Driven Macro/Meso/Micro/Nano Porous Evolution

**Figure 1:**
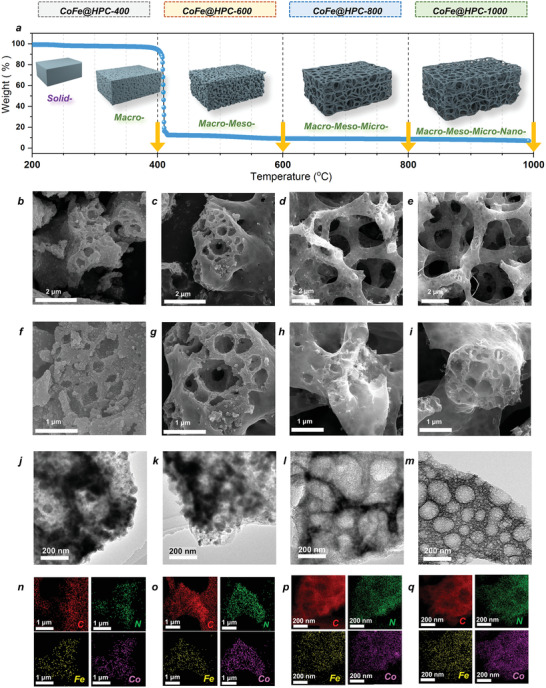
The TGA curve of CoFe@E‐MTF (ground layer in (a)) and the 3D illustration (upper layer in (a)), SEM (b–i), TEM (j–m), EDS mapping (n–q) images for CoFe@HPC*x* (*x* = 400, 600, 800, and 1000).

To incorporate energetic functional organic fragments into the precursor, 1H‐1,2,3‐triazole ligand was employed as a building block to assemble the crystalline CoFe@E‐MTF. The morphology of E‐MTF and CoFe@E‐MTF crystals were observed by scanning electron microscope (SEM) and transmission electron microscope (TEM), displaying smooth octahedrons with an average diameter of ≈150 nm (Figures S1 and S2, Supporting Information). The greatly matched simulated and experimental powder X‐ray diffraction (PXRD) curves, and the uniform distribution of corresponding Co, Fe, C, N, and O elements in energy dispersive spectroscopy (EDS) mapping indicated the highly‐ordered arrangement of metals and N_3_‐bonds in crystalline structures of E‐MTF and CoFe@E‐MTF (Figure S3, Supporting Information). As thermal gravimetric analysis (TGA) shown in **Figure** **1**a, the fast breakage of energetic N_3_‐bonds at ≈420 °C, resulted in the high detonation velocity and pressure in the decomposition of CoFe@E‐MTF. Such a rapid in situ volume expansion and mass loss process generated a kind of foam‐like hierarchical porous carbon with ultra‐low density above 420 °C.

We captured four products under different calcination temperatures of 400, 600, 800, and 1000 °C (Figure 1a), respectively, denoted as CoFe@HPC*x* (*x* = 400, 600, 800, and 1000). The residual mass of ≈10%, 9%, and 8% for CoFe@HPC600, CoFe@HPC800, and CoFe@HPC1000 were compared to their volume, presenting a high volume‐mass ratio (*V*/*M*, the volume of 1 g pristine CoFe@E‐MTF as one equivalent volume, Figure S4, Supporting Information) of ≈1.43, 80, 105.6, 125. For further study of the porous morphology and structural evolution process, the SEM and TEM images of CoFe@HPC*x* are presented in Figure 1b–m and Figures S5‐S12, Supporting Information. The porous structures showed significant differences with the increase in temperature. The distribution of macro‐pores (beyond 200 nm), macro/mesopores (beyond 20 nm), macro/meso/micro‐pores (beyond 2 nm), and macro/meso/micro/nano‐pores (beyond 1 nm) for CoFe@HPC400, CoFe@HPC600, CoFe@HPC800, and CoFe@HPC1000 were observed. The ordered hierarchical structures under high temperatures (800 and 1000 °C), especially many sub‐nano porous structures with clear boundaries for CoFe@HPC1000 were observed in high‐resolution TEM images (Figure S12, Supporting Information).

More importantly, the volume expansion and structural stretching of carbon matrix for energetic precursor at high temperature were utilized for homogeneous planting sub‐nano Co/Fe cluster or single atom in the minimum quantity. The EDS mapping images (Figures S9–S12, Supporting Information) indicated the uniform dispersion of Co and Fe atoms in the CoFe@HPC*x* series. By incorporating hierarchical porous features and uniformly dispersed magnetic metals, this system achieves both lightweight property with high metal atom utilization, as well as the highly efficient propagation 3D network for electromagnetic wave attenuation through multiple reflections and scattering.

### 2.2 Regulation of EW Absorbing Intensity and Bandwidth

**Figure 2:**
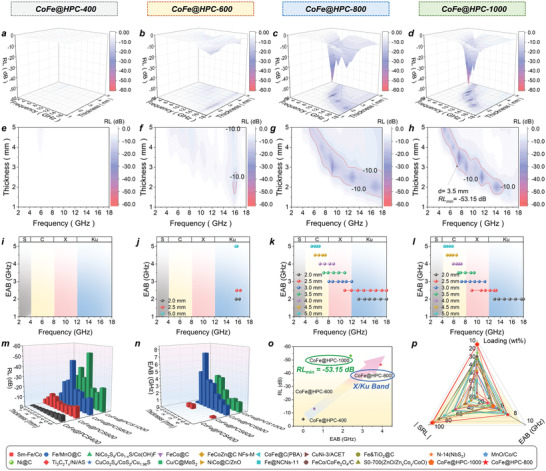
The 3D RL values (a–d), 2D RL contour map (e–h), EAB (i–l), comparison of RL values (m), and EAB (n) in different thickness (m,n), summary and comparison of RL and EAB (o) for CoFe@HPC*x* (*x* = 400, 600, 800, and 1000), comparison of CoFe@HPC800 and CoFe@HPC1000 with reported EWAs in literature (p).

The electromagnetic wave absorbing behaviors of the CoFe@HPC*x* series were evaluated by 3D *RL*, 2D *RL* contour map, and effective absorption bandwidth (EAB) (*RL* < −10 dB) at the range of ≈2–18 GHz under a filler loading of 15 wt%. Poor EW adsorptions for low‐temperature calcinated samples were observed, as shown in **Figure** **2**, the *RL*_min_ of CoFe@HPC600 reaches only −13.14 dB with a narrow EAB of 0.56 GHz at the thickness of 2 mm, and the *RL*_min_ of CoFe@HPC400 could not even reach −10 dB. The PXRD of the CoFe@HPC*x* series indicated the characteristic peaks of 34.6°, 36.4°, 63°, and 67.9° for (102), (020), (222), and (024) crystal planes of Fe_3_C (PDF# 03–0400) and 43° and 57.1° for (111) and (112) planes of Co_2_C (PDF# 50–1371) in CoFe@HPC400 and CoFe@HPC600, implying the insufficient reduction of metals in the composites. The distinct peaks of 19.2°, 28.1°, and 28.6° in CoFe@HPC400 are attributed to the maintained crystalline peaks of undecomposed CoFe@E‐MTF precursor. The enhanced broad peak of ≈22.4° for CoFe@HPC800 and CoFe@HPC1000 can be indexed to (002) planes of graphite carbon, indicating the increased degree of graphitization at high temperatures. As a result, CoFe@HPC800 showed an intense *RL*_min_ of −46.40 dB and wide EABs of 4 (≈8–12 GHz) and 7.5 GHz (≈10.5–18 GHz) at 3 and 2.5 mm, which fully covered X and Ku bands, respectively. The CoFe@HPC1000 exhibited an excellent performance on EW absorbing intensity, displaying the highest *RL*_min_ of −53.15 dB at 3.5 mm. It is worth noting that, considering the low density (*V*/*M*) and only 15 wt% loading content for CoFe@HPC*x* fillers, the superior *SRL* of −101.24 dB mg^−1^ mm^−1^ for CoFe@HPC1000 far exceeded the reported EWAs in literature. The dual‐regulation of EW absorbing intensity and bandwidth on an ultralight hierarchical porous carbon was achieved, showing promising commercial applications.

### 2.3 Interfacial and Vortical Dipole in Graphitized Matrix

**Figure 3:**
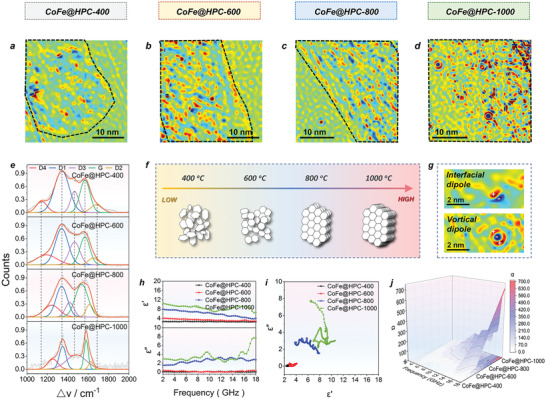
The charge density maps (a–d), deconvolution peaks in Raman spectra (e), structural illustration images of graphitization degree (f), the local observed interfacial dipole and vortical dipole (g), the real part and imaginary part of permittivity (h), the Cole–Cole plots (i), and the 3D attenuation coefficient (j) for CoFe@HPC*x* (*x* = 400, 600, 800, and 1000).

The CoFe@HPC*x* (*x* = 400, 600, 800, and 1000) is a series of dielectric‐dominated EW absorbing materials. According to the relative complex permittivity equation of *ε*_r_ = *ε*′ *– jε″*, the real part (*ε′*) and the imaginary part (*ε″*) represent electric storage and dissipation capacity, respectively, which are highly related to the conductivity and relaxation polarization. The sp^2^ hybrid carbon sites and the distorted/defective carbon sites in CoFe@HPC*x* were fitted and analyzed by typical D1 (≈1340 cm^−1^) and G (≈1560 cm^−1^) peaks in Raman spectra (**Figure** **3**e and Figure S14 and Table S1, Supporting Information). Compared with the *I*_D1_/*I*_G_ ratios for CoFe@HPC400 (1.10) and CoFe@HPC600 (1.11), the lower *I*_D1_/*I*_G_ ratios (0.93 and 0.82) and the intense G peaks for CoFe@HPC800 and CoFe@HPC1000 indicated the improved graphitization degrees at the high temperature (Figure 3f), which are in accordance with the PXRD results (Figure S13, Supporting Information). In Figure 3h and Figure S15a, Supporting Information, the highest of *ε′*, *ε″*, and tan *δ*_ε_ values for CoFe@HPC1000 implied the superior electrical conductivity and electron movements, contributing to the conversion from EW to thermal energy.

The relaxation polarization of CoFe@HPC*x* could be evaluated by dipole and interfacial polarization, which can be illustrated by Debye's theory (Equations (S8–S10), Supporting Information). The Cole–Cole semicircles (Figure 3i and Figure S16, Supporting Information) among *ε′* and *ε″* can be used to explain the ability of polarization loss, displaying a sequence of CoFe@HPC1000 > CoFe@HPC800 > CoFe@HPC600 > CoFe@HPC400. The highly distorted Cole–Cole curve of CoFe@HPC1000 indicated a large number of disordered atoms or defects. Hence, combining both conductivity and polarization effects in the CoFe@HPC*x* series (Equation (S11), Supporting Information), the highest attenuation constant (*α*) for CoFe@HPC1000 in the sequence of CoFe@HPC1000 (704.44) > CoFe@HPC800 (390.41) > CoFe@HPC600 (199.17) > CoFe@HPC400 (37.39) was demonstrated.

The structural origins of this polarization in CoFe@HPC*x* were investigated by calculated R2 values (Equation (S13) and Table S1, Supporting Information) in Raman spectra, which reflected the number of structural defects. The R2 value of 0.38 for CoFe@HPC1000 revealed its highest number of defects in the carbon matrix, which can promote dipole polarization. The highest peak area of the D3 band (*A*_D3_ of 80.4 in Table S1, Supporting Information) which is attributed to the disordered carbon confirmed the transformation of numerous nanocrystalline graphite in CoFe@HPC1000. Hence, it can be concluded that carbon atoms in such a hierarchical porous architecture existed as numerous tiny crystalline domains with sp^2^ hybridization, however, in a highly disordered and randomly stacked manner.^[^ ^17^ ^]^

The polarization wall/edges for the diverse scale of pores in the CoFe@HPC*x* matrixes was investigated by the HR‐TEM and hologram (Figure 3a–d), reflecting different scales of charge density distribution maps. In the map, red represents the positive charge, blue represents the negative charge, and the lightness color represents relative intensity (Figure S17, Supporting Information). The non‐uniform large polarized charge regions in CoFe@HPC400 were detected (Figure 3a), which could be attributed to the insufficient graphitization and porosity. The increased temperature induced the homogenous distribution of micro‐ and nano‐pores in CoFe@HPC800 and CoFe@HPC1000, giving the high‐density distribution of nanoscale charge dipoles (≈1–3 nm). A large number of dense nanoscale interfacial polarization was generated in CoFe@HPC800 (Figure 3g, upper). The intense dark red and blue charge dipoles in CoFe@HPC1000 revealed the local charge enrichment (Figure 3d), which confirms the highest degree of polarization. Moreover, to our surprise, many unique vortical dipoles with a diameter of ≈1.2–1.5 nm were founded in CoFe@HPC1000 (Figure 3g, under), which have never been detected before. The vortex dipoles are well matched with the diameter of ≈1–1.5 nm nanopores in TEM images of CoFe@HPC1000. Besides the defects and dipoles on the high porous matrix, the existing status of discrete distribution of Co and Fe metals may play important roles, which should be investigated in depth.

### 2.4 Electron‐Driven Co─Fe Coupling in Vortical Magnetic Nano‐Domains

**Figure 4:**
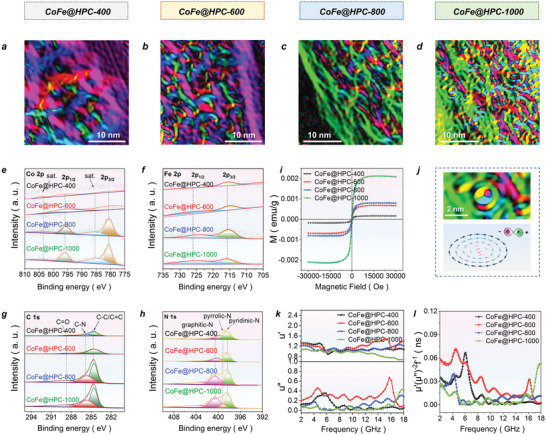
The magnetic moment distribution maps (a–d), the XPS spectra of Co 2p (e), Fe 2p (f), C 1s (g), and N 1s (h), the magnetic hysteresis loops (i), the illustration images of vortex magnetic domains and spin texture (j), the real part and imaginary part of permeability (k), and the *C*_0_ value (l) for CoFe@HPC*x* (*x* = 400, 600, 800, and 1000).

Only about ≈0.67–1.18 wt% Co and Fe (Table S2, Supporting Information) were highly dispersed into the carbon matrix in the CoFe@HPC*x* series. The chemical valent states of all the elements in CoFe@HPC*x* are analyzed by XPS (**Figure** **4**e–h). The highly discrete Co and Fe metals in CoFe@HPC800 and CoFe@HPC1000 resulted in the enhanced low valent binding energy peaks at 780.6/795.9 eV for Co 2p and at 715.7/726 eV for Fe 2p (Figure 4e,f). The growing intense peaks for C─C/C═C and C─N at 284.60 and 285.82 eV in C 1s spectra and graphitic N at 400.52 eV in N 1s spectra are attributed to the improved graphitization degrees in CoFe@HPC800 and CoFe@HPC1000 (Figure 4g,h). Moreover, the higher pyridinic N and pyrrolic N peaks at 398.32 and 399.17 eV represented the more dipole polarization centers on the carbon matrix (Figure 4h).

The existing status of the Co and Fe metals influences the saturation magnetization of the material, which reflects the overall magnetic effects and associates with the permeability (*µ*_r_ = *µ′ − jµ*″) and magnetic loss (tan *δ*_µ_ = *µ″*/*µ*′). Generally, the *µ′*, *µ″*, and tan *δ*_µ_ values are running at low levels (Figure 4k and Figure S15b, Supporting Information), indicating the relatively small magnetic loss contributions. In addition, the non‐negligible eddy current loss of CoFe@HPC*x* can be evaluated by coefficient value (*C_0_* = *µ″*(*µ′*)^−2^f^−1^), which laid below 0.1 ns at the whole frequency range of ≈2–18 GHz (Figure 4l), indicating the suppressed eddy current effect. As Figure 4i shown, the calculated saturation magnetization (*M*_s_) values for CoFe@HPC*x* (*x* = 400, 600, 800, and 1000) are 1.56 × 10^−4^, 6.87 × 10^−4^, 8.12 × 10^−4^, and 21.3 × 10^−4^ emu g^−1^, respectively. The increased *M*_s_ from CoFe@HPC400 to CoFe@HPC600 and CoFe@HPC800 is ascribed to the crystallization and reduction of Co and Fe metals, which provide the ordered magnetic domains. In CoFe@HPC1000, the highly dispersed Co/Fe atoms on discontinuous nanopores and their coupling interaction gave the highest *M*_s_ value and the formation of high‐density magnetic nano‐domains. The coercivity (*H*_c_) values are highly related to the number of defects in this series, therefore, the lowest *H*_c_ of 47.06 Oe was observed.

The LTEM holography images displayed the multi‐domain structure of the CoFe@HPC*x* (Figure 4a–d). More complex domain walls near the smaller pores were observed, manifesting the lowest local free energy under the porous shape confinement. As the heat treatment temperature increases, the magnetic domains become more complex with the discontinuous distribution of magnetic moments. Hence, CoFe@HPC1000 showed the highest demagnetizing energy and exchange energy, and its dynamic magnetic domains corresponded to intense magnetic energy dissipations. In CoFe@HPC1000, the shifts and overlayers of domain walls generated the vortex magnetic domains which can be schematically described as spin textures (Figure 4j) and has never been reported in the porous EWAs as far as we know.^[^ ^18^ ^]^

### 2.5 Single‐Atomic Evolution of Co and Fe during the Calcination

**Figure 5:**
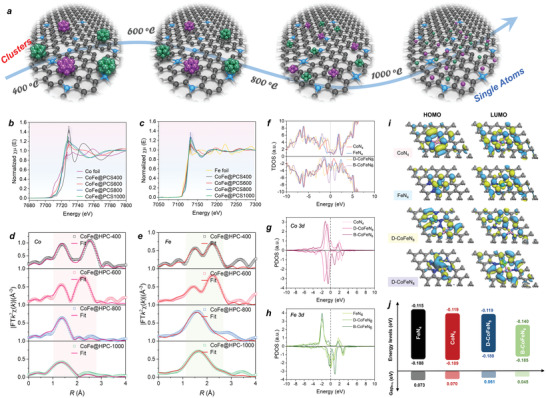
The illustration of Co and Fe existing status and during the structural evolution process under calcination (a), the experimental XANES spectra (b,c), the *K*‐edge EXAFS spectra (d,e), the theoretically calculated TDOS curves (f), the PDOS curves (g,h), the LUMO and HOMO images (i), and the orbital energy levels and band gaps for CoFe@HPC*x* (*x* = 400, 600, 800, and 1000).

The generation of magnetic domains is directly related to the distribution form and electronic structure of Co and Fe metals on the matrix, which was clarified by XANES and *K*‐edge EXAFS spectroscopy. The XANES (**Figure** **5**b) displays the lowest Co valence of CoFe@HPC1000 close to that of Co foil. The highest Co valence in CoFe@HPC400 is attributed to the insufficient reduction of metals. The valent state of Co in CoFe@HPC600 and CoFe@HPC800 lies between CoFe@HPC400 and CoFe@HPC1000. In Figure 5c, the valence of Fe in CoFe@HPC*x* gradually increases in slight degrees with the increase of their treatment temperature, indicating the charge transfers between Fe and Co. The Fourier transform of *K*‐edge EXAFS (Figure 5d,e and Table S3, Supporting Information) indicated that most of Co and Fe initially exist as clusters (Co─Co bonds around ≈2.50–2.86) at low temperatures and gradually split into single atoms (Co─N bonds around ≈1.87–2.04) as temperature rise in CoFe@HPC*x* series (Figure 5a). The wavelet‐transformed images for the *k* ^2^‐weighted EXAFS (Figures S24 and S25, Supporting Information) clearly displayed the metallic clusters and single atoms in CoFe@HPC*x* on radial distance maps.

For revealing the internal structure diversities and their EW attenuation mechanism, the density functional theory (DFT) theoretical calculations based on the isolated CoN_4_, FeN_4_, discrete CoFeN_8_ (D‐CoFeN_8_), and bonding CoFeN_6_ (B‐CoFeN_6_) models (Figure S26, Supporting Information) were conducted. As shown in Figure 5f, the total density of states (TDOS) curves displayed the stronger TDOS peaks lay closer to the Fermi level for D‐CoFeN_8_ and B‐CoFeN_6_ than those for CoN_4_ and FeN_4_, indicating the higher energy band overlaps in D‐CoFeN_8_ and B‐CoFeN_6_. In addition, the partial density of states (PDOS) of the Co 3d and Fe 3d (Figure 5g,h) showed higher spin polarization in D‐CoFeN_8_ and B‐CoFeN_6_, indicating the half‐metallic property and magnetic interaction among Co and Fe. The lowest unoccupied molecular orbital (LUMO) and highest occupied molecular orbital (HOMO) for the four models indicated the gradually enriched orbitals around Fe atoms by reducing the Co─Fe distance (Figure 5i). Therefore, compared with the band gaps for CoN_4_ (0.073 eV), FeN_4_ (0.070 eV), and D‐CoFeN_8_ (0.061 eV), the lowest band gap of 0.045 eV is observed for D‐CoFeN_6_ (Figure 5j) confirmed its enhanced Co─Fe charge transfer, which is accordance with the XANES results (Figure 5b,c).

### 2.6 Synergy of Multi‐Reflection with Nano‐Confinement in Hierarchical Architecture

**Figure 6:**
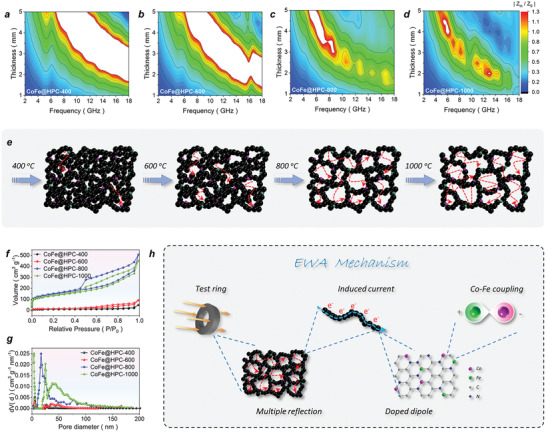
The 2D contour maps |*Z*_in_/*Z*_0_ | (a–d), the illustration of multiple scatterings (e), the N_2_ adsorption–desorption isotherms (f), the pore size distribution maps (g) for CoFe@HPC*x* (*x* = 400, 600, 800, and 1000), the scheme of EW absorbing mechanism (h).

To pursue a deeper insight into the EW absorbing behavior, the EW reflection of the CoFe@HPC*x* series was evaluated by 2D impedance matching contour maps of |*Z*_in_/*Z*_0_| (**Figure** **6**a–d and Figure S18, Supporting Information). In Figure 6c, the well‐matched |Z_in_/Z_0_| close to 1 for CoFe@HPC800 at ≈8–18 GHz is observed, meaning the incident wave permeates the absorber at the greatest degree with less reflection. It is the main reason for the wide EAB (fully covered X/Ku band) of CoFe@HPC800. The matched |*Z*_in_/*Z*_0_| for CoFe@HPC1000 at the frequency around 7.52 GHz delivered the strongest *RL*_min_ of −53.15 dB. In contrast, the big white areas in the maps of CoFe@HPC400 and CoFe@HPC600 indicate the severely mismatched impedance (Figure 6a–d), which should be attributed to the relatively lower porosities. Compared with the superior high Brunauer–Emmett–Teller (BET) specific surface areas of 206.75 and 370.73 m^2^ g^−1^ for CoFe@HPC800 and CoFe@HPC1000 in the N_2_ adsorption–desorption isotherms and pore diameter distribution maps (Figure 6f,g), the low BET specific surface areas of 29.56 and 51.04 m^2^ g^−1^ for CoFe@HPC400 and CoFe@HPC600 are detected. As illustrated in Figure 6e, the increased porosity and diverse pore diameters distributed in the whole macro/meso/micro/nano‐porous range for the high‐temperature treated samples resulted in enhanced multiple internal EW scattering. Combined with the superior electric conductivity, nano‐vortex polarization, and magnetic exchange energy, the dual‐function regulation of EW absorbing intensity and bandwidth is successfully realized in an ultralight molecular carbon sponge (Figure 6h).^[^ ^19^ ^]^

## 3 Discussion

The structural design strategies of CoFe@HPC*x* are concluded in three aspects: i) Design and assemble an atypical hierarchical macro/meso/micro/nano‐pores with high EW multiple scatterings. Energetic triazole as a ligand was implanted into the crystalline energetic MOF for driving rapid explosion and volume expansion owing to the N═N─N bonds. ii) Constructing high‐density of nanoscale interfacial/vortical polarization. The rapid stretching of carbon substrate with the improved heat‐treated temperature during the calcination induced the enhanced graphitization degree and increased number of disorders/defects. iii) Building vortical magnetic nano‐domains with high‐dynamic demagnetizing energy/exchange energy. Adjustable atomic Co─Fe coupling sites inserted on the walls of carbon matrix nanopores in high dispersion manner resulting in the formation of vortical magnetic domains. iv) The energy band overlaps promote the charge transfer of Co─Fe units with the enhancement of the density of states. Capturing and monitoring the nano‐porous structural evolution and single‐atom metallic transformation by XANES, EXAFS, LTEM, and hologram imaging contributed to explaining the formation of spin polarization and magnetic exchange through PDOS analysis in DFT theoretical calculations.

## 4 Conclusion

In summary, we uniformly planted high‐energetic N_3_‐bonds into a CoFe‐bimetallic energetic metallic triazole framework (CoFe@E‐MTF) precursor under a facile room‐temperature reaction. Utilizing the rapid volume expansion process caused by the decomposition of energetic bonds, we successfully captured four atomic CoFe‐inserted hierarchical porous carbon (CoFe@HPC*x*, *x* = 400, 600, 800, and 1000 °C) with diverse nano‐porous structures and metallic status under different calcinated temperature. In the CoFe@HPC*x* series, the chemical bonding and existence status of Co and Fe in materials was deeply analyzed by XANES and EXAFS spectra, revealing the structural evolution of Co and Fe metal from the cluster to single atom state on the carbon matrix. The LTEM and hologram imaging in situ monitor the formation and distribution of high‐density nano‐vortex dipoles and magnetic domains on a 3D conductive network, contributing to revealing the EW attenuation mechanism of Co─Fe coupling dipoles. Under the filler loading of 15 wt%, the bifunctional regulation of fully covered X and Ku bands at 3 and 2.5 mm for CoFe@HPC800 and the superb *RL*_min_ of −53.15 dB and SRL of −101.24 dB mg^−1^ mm^−1^ for CoFe@HPC1000 were achieved. This work highlights an atomic‐level structure editing strategy for assembling dielectric/magnetic synergetic ultra‐light EWAs with dual‐function regulation.

## Conflict of Interest

The authors declare no conflict of interest.

## Supporting information

Supporting Information

## Data Availability

The data that support the findings of this study are available from the corresponding author upon reasonable request.
