# Supplementary material for: High‐Density Nanopore Confined Vortical Dipoles and Magnetic Domains on Hierarchical Macro/Meso/Micro/Nano Porous Ultra‐Light Graphited Carbon for Adsorbing Electromagnetic Wave
Source: Adv Sci (Weinh). 2023 Aug 1;10(28):2303217. doi: 10.1002/advs.202303217 (PMC10558675; doi:10.1002/advs.202303217)
Supplement: Supplementary file 1 — Supporting Information [file ADVS-10-2303217-s001.pdf]

ADVANCED SCIENCE
Open Access

Supporting Information

for *Adv. Sci.*, DOI 10.1002/advs.202303217

High-Density Nanopore Confined Vortical Dipoles and Magnetic Domains on Hierarchical Macro/Meso/Micro/Nano Porous Ultra-Light Graphited Carbon for Adsorbing Electromagnetic Wave

Wenhuan Huang, Xingxing Zhang, Jiamin Chen, Qiang Qiu, Yifan Kang, Ke Pei, Shouwei Zuo, Jincang Zhang and Renchao Che**

Supporting Information

High-density Nanopore Confined Vortical Dipoles and Magnetic Domains on Hierarchical Macro/Meso/Micro/Nano Porous Ultra-Light Graphited Carbon for Adsorbing Electromagnetic Wave

Wenhuan Huang,† Xingxing Zhang,† Jiamin Chen, Qiang Qiu, Yifan Kang, Ke Pei, Shouwei Zuo, Jincang Zhang and Renchao Che**

† These author contributed equally to this work.

Dr. J. Chen, Dr. X. Zhang, Mr. Q. Qiu, Dr. Y. Kang, Dr. S. Zuo, Prof. W. Huang.

Key Laboratory of Chemical Additives for China National Light Industry, College of Chemistry and Chemical Engineering, Shaanxi University of Science and Technology, Xi'an 710021, China.

Email: huangwenhuan@sust.edu.cn

Dr. K. Pei, Prof. R. Che.

Laboratory of Advanced Materials, Shanghai Key Lab of Molecular Catalysis and Innovative Materials, Academy for Engineering & Technology, Fudan University, Shanghai 200438, P. R. China.

Email: rcche@fudan.edu.cn

Prof. J. Zhang, Prof. R. Che.

Zhejiang Laboratory, Hangzhou 311100, P. R. China

TABLE OF CONTENTS

1. Experimental Section 1

2. Characterizations 2

3. Data analysis 3

4. Results and discussions 6

References 36

1. Experimental Section

1.1. Synthesis of E-MTF

First, $ZnCl_2$ (5.0 g) was dissolved in a solvent mixture of ethanol (50 mL), water (75 mL), ammonium hydroxide (25%-28%, 20 mL) and N, N-dimethylformamide (DMF, 50 mL), and then kept stirring for 10 min. Next, 1H-1,2,3-triazole (6.26 mL) was slowly dripped in the solution during stirring. After 24 h stirring at room temperature, the white product was washed with by ethanol, collected by filtration and dried at 80 ℃ for 8 h with a yield of 90 %.

1.2. Synthesis of CoFe@E-MTF

For synthesizing CoFe@E-MTF, $Co(CH_3COO)_2·6H_2O$ (0.72 g) and $FeCl_2$ (0.49 g) in a molar ratio of 1:1 were first dissolved in 200 mL methanol. Next, E-MTF (2.0 g) powder was immersed into above solution, and then stirred at room temperature for 6 h. The pink powder was filtered out and washed by ethanol for three times. After the vacuum drying at 100 ℃ for 8 h, the CoFe@E-MTF was collected with a yield of 62%.

1.3. Synthesis of CoFe@HPCx (x=400, 600, 800, 1000)

The as-prepared 2.0 g CoFe@E-MTF was put into the ceramic boat and then placed in the programmed tube furnace. It was heated up to 1000 ℃ at a heating rate of 5 ℃/min^{-1} under the nitrogen atmosphere. After that, the furnace was kept at 1000 ℃ for 2 h and then naturally cooled to room temperature. The ultra-light black powder of CoFe@HPC-1000 (0.16 g) was successfully synthesized, It was heated up to 400, 600, 800 ℃ at a heating rate of 5 ℃/min^{-1} under the nitrogen atmosphere, synthesized CoFe@HPC-400, CoFe@HPC-600, CoFe@HPC-800.

2. Characterizations

D8 DAVANCI X-ray powder diffractometer equipped with graphite monochromatized Cu Kα radiation (λ = 0.1542 nm) was used to record powder X-ray diffraction (PXRD) patterns in the 2θ range of 5°-80° with a scanning rate of 1 ^{o}/min. Scanning electron microscopy (SEM) images were collected by a Hitachi S4800 apparatus with an acceleration voltage of 2 kV. The transmission electron microscopy (TEM) images were recorded on a JEM-2010HR apparatus working at an accelerating voltage of 200 kV and X-ray energy-dispersive spectroscopy (EDS) was taken on a JEM-2010HR-Vantage typed energy spectrometer. X-ray photoelectron spectroscopy (XPS) was implemented on Thermo ESCA Lab250XI. The surface area (BET) of the samples was measured by N_2 adsorption and desorption at 77K using an ASAP 2020 sorption system. Raman spectroscopy of the samples was obtained by a Renishaw in Via Raman Microscope. The electromagnetic parameters were analyzed using a HP8753D vector network analyzer in the frequency range of 2-18 GHz. The measured samples were dispersed in paraffin homogeneously with a sample-to-paraffin weight ratio of 3:17, and then the mixture was pressed into a toroidal shape with an inner diameter of 3.04 mm and an outer diameter of 7.0 mm. The hysteresis loop of the materials was tested by superconducting quantum interference device MPMS(SQUID) VSM magnetometer. Off-axis electron holography was performed on a Lorentz transmission electron microscope (JEOL JEM-2100F). The absorption spectra of Mo-edge were collected in a transmission mode at room temperature using a Si (111) double crystal monochromator at the 1W1B station of Beijing Synchrotron Radiation Facility (BSRF, Beijing).

3. Data analysis

The reflection loss (RL) values of the absorbers are calculated according to transmission line theory by the following equation S1-2:

$$RL(dB) = 20lg \mid \frac{Z_{in} - Z_0}{Z_{in} + Z_0} \mid \qquad \text{(Equation S1)}$$

$$Z_{in} = Z_0 \sqrt{\frac{\mu_r}{\varepsilon_r}} \tanh\left[j\left(\frac{2\pi f d}{c}\right)\sqrt{\mu_r \varepsilon_r}\right] \qquad \text{(Equation S2)}$$

Where Z_0 is the characteristic impedance of free space, Z_{in} is the normalized input impedance of absorber, ε_r and μ_r are the relative complex permittivity and permeability, d is the layer thickness, c is the speed of light in free space and f is the frequency.

The absorption peak shifts toward low frequency with increasing the thickness, which can be explained by Equation S3 according to quarter-wavelength matching model**:**

$$d_m = \frac{n\lambda}{4} = \frac{nc}{4 f_m \sqrt{|\varepsilon_r||\mu_r|}} \qquad (n = 1,3,5, \ldots) \qquad \text{(Equation S3)}$$

The d_m and f_m in the equation represent the matching thickness and the matching frequency, respectively. The interference effect between the reflected and incident microwave will result in the microwave energy dissipation when the d_m and f_m value accords with Equation S3.

The practical application of absorbing materials is calculated by Equation S4.

$$SRL = \frac{RL_{\min}}{\text{Thickness} \times \text{Loading}} \qquad \text{(Equation S4)}$$

Cole–Cole semicircle model (Equation S5):

$$(\varepsilon' - \frac{\varepsilon_{s+}\varepsilon_{\infty}}{2})^2 + (\varepsilon'')^2 = (\frac{\varepsilon_{s-}\varepsilon_{\infty}}{2})^2 \qquad \text{(Equation S5)}$$

Each semicircle in the ε′–ε″ curve stands for a polarization relaxation process. The ε_s and ε_∞ represent the static dielectric constant, the dielectric constant at infinite frequency, respectively. The high number of semicircles means the strong dipole polarization process.

Debye relaxation correction formula (Equation S6-7):

$$\varepsilon_r = \varepsilon_{r\infty} + \frac{\varepsilon_{rs} - \varepsilon_{r\infty}}{1 + (i\omega\tau)^{1-A}} \quad (0 < A < 1) \qquad \text{(Equation S6)}$$

$$\varepsilon_r' = \varepsilon_{r\infty} + (\varepsilon_{rs} - \varepsilon_{r\infty}) \frac{1 + (\omega\tau)^{(1-A)} sin\frac{\pi A}{2}}{1 + 2(\omega\tau)^{1-A} sin\frac{\pi A}{2} + (\omega\tau)^{2(1-A)}} \qquad \text{(Equation S7)}$$

ε_p'' and ε_c'' are the dielectric loss contributed by polarization relaxation and charge transport, respectively, which can be obtained according to Debye theory (Equation S8-10).

$$\varepsilon_c'' = \frac{\sigma}{2\pi f \varepsilon_0} \qquad \text{(Equation S8)}$$

$$\varepsilon_p'' = \frac{\varepsilon_s - \varepsilon_\infty}{1 + (2\pi f)^2 \tau^2} \omega\tau = \varepsilon'' - \varepsilon_c'' \qquad \text{(Equation S9)}$$

$$\varepsilon" = \frac{\varepsilon_s - \varepsilon_\infty}{1 + (2\pi f)^2 \tau^2} \omega\tau + \frac{\sigma}{2\pi f \varepsilon_0} = \varepsilon_p'' + \varepsilon_c'' \qquad \text{(Equation S10)}$$

Where ε_s is the relative permittivity at static, and ε_∞ is that at “infinite” high frequency. σ is the conductivity, Conductivity is a parameter used to describe the difficulty of charge flow in matter.

The attenuation coefficient (α):

$$\alpha = \frac{\sqrt{2}\pi f}{C} \sqrt{(\mu''\varepsilon'' - \mu'\varepsilon') + \sqrt{(\mu''\varepsilon'' - \mu'\varepsilon')^2 + (\mu'\varepsilon'' + \mu''\varepsilon')^2}} \qquad \text{(Equation S11)}$$

$$C_0 = \mu"(\mu')^{-2} f^{-1} = 2\pi\mu_0 d^2 \sigma \qquad \text{(Equation S12)}$$

The *R2* value reflects the graphitization and the number of structural defects and assumes value

higher than 0.5 for poorly organized structure and lower than 0.5 for a well-organized one, which can be calculated by the following formula (Eq. S13):

$$R2 = \frac{I_{D1}}{I_G + I_{D1} + I_{D2}} \qquad \text{(Equation S13)}$$

The band intensity ratio can be used to determine the degree of the graphitization, which can be calculated according to equation S14:

$$\frac{I_{D1}}{I_G} = C'(\lambda) d_{nc}^{-2} \qquad \text{(Equation S14)}$$

where C′ (514 nm) ≈ 0.0055 Å^{-2}.

4. Results and discussions

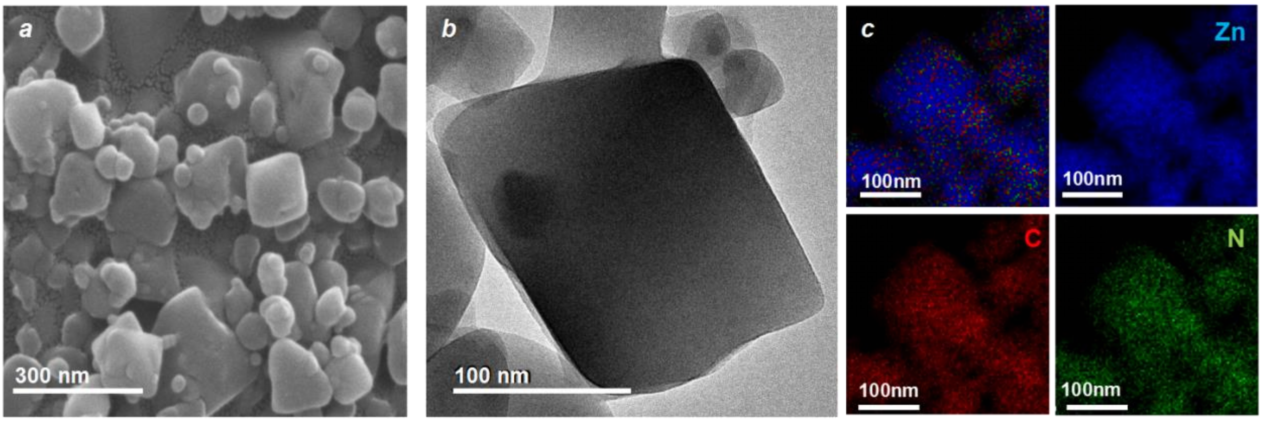

Figure S1. a) the SEM, b) TEM and c) TEM-EDS mapping of E-MTF.

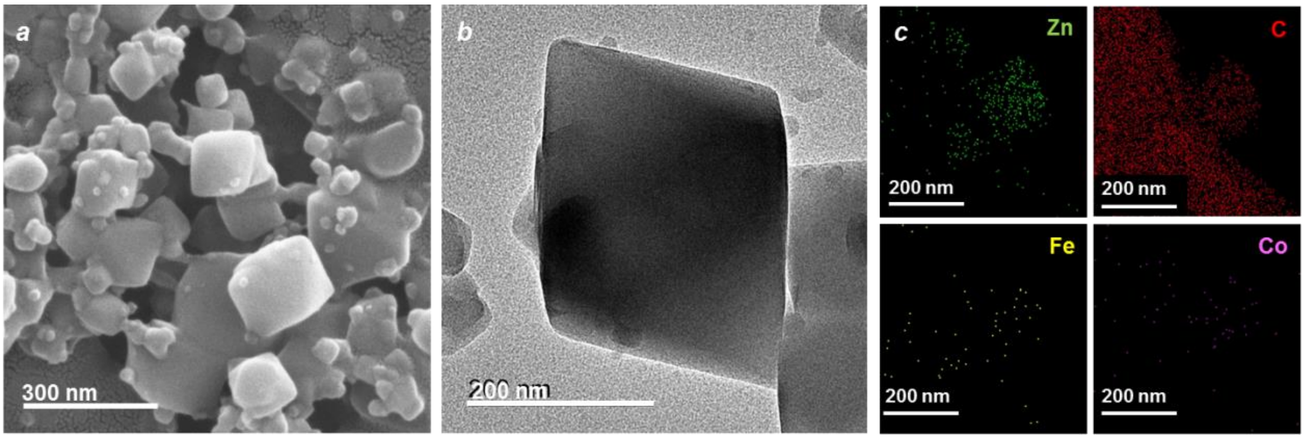

Figure S2. a) the SEM, b) TEM and c) TEM-EDS mapping of CoFe@E-MTF.

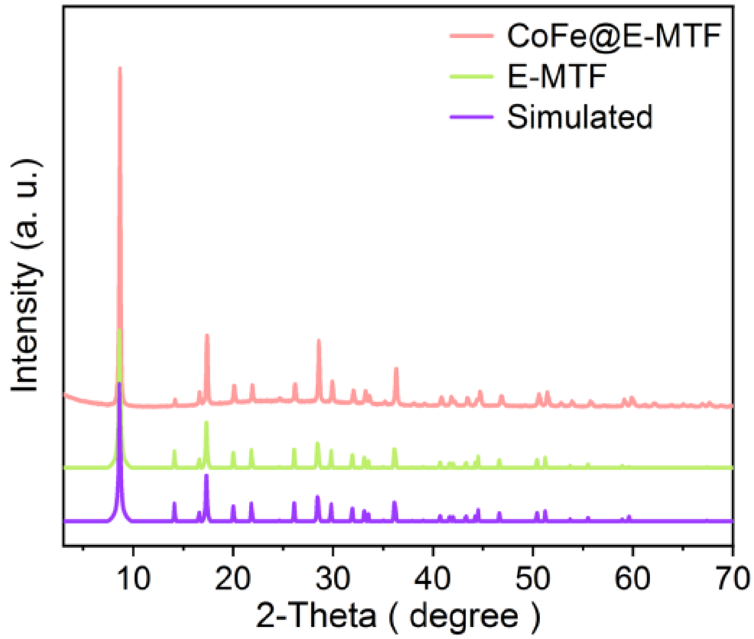

Figure S3. The PXRD results of synthesized CoFe@E-MTF，E-MTF and the simulated.

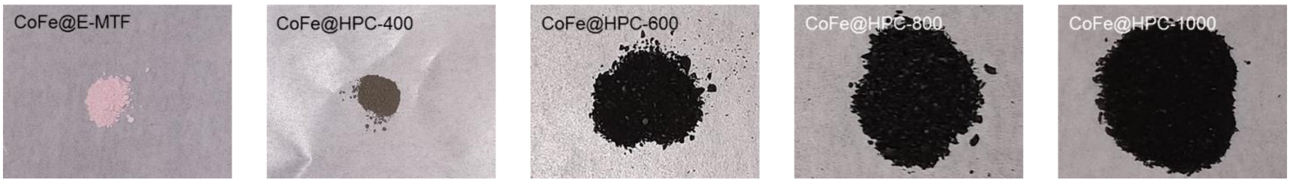

Figure S4. The photography of CoFe@E-MTF and CoFe@HPC-x (x = 400, 600, 800 and 1000)

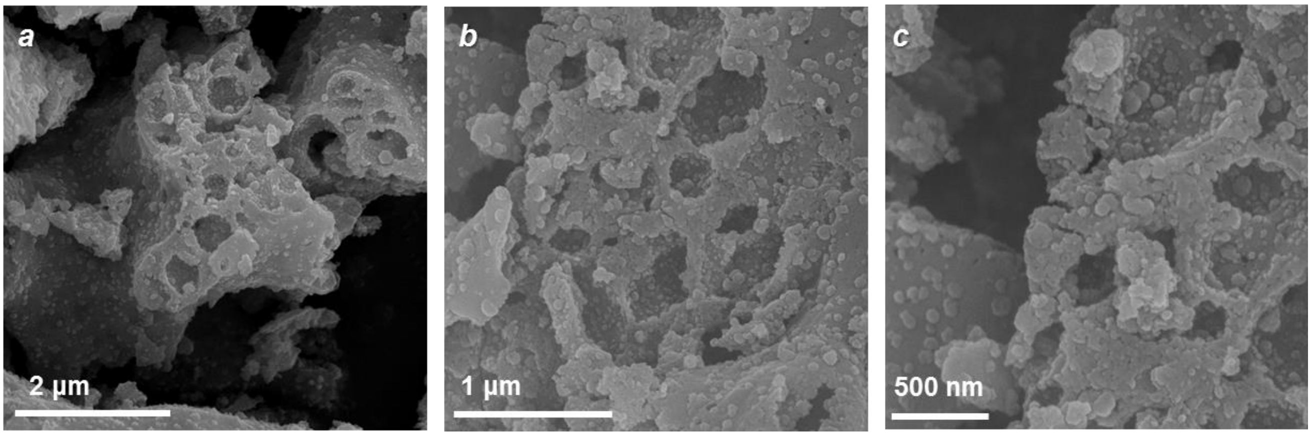

Figure S5. a-c) the SEM images of CoFe@HPC-400.

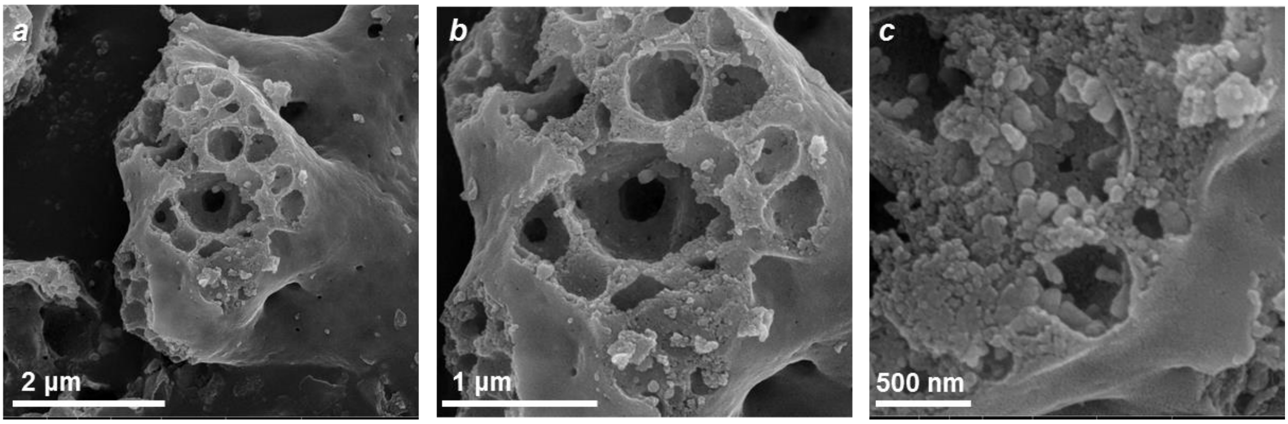

Figure S6. a-c) the SEM images of CoFe@HPC-600.

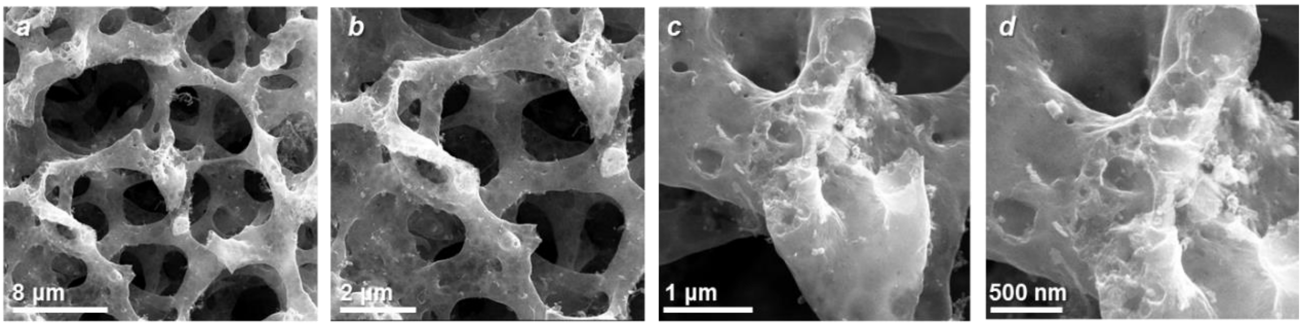

Figure S7. a-d) the SEM images of CoFe@HPC-800.

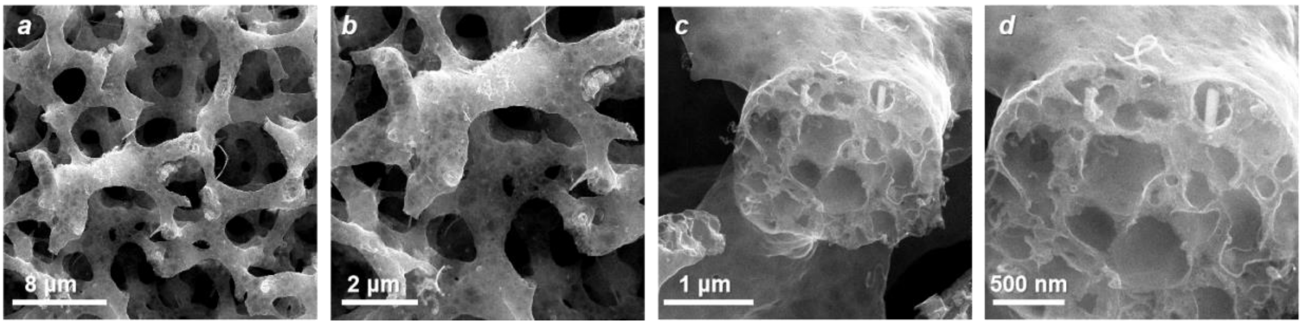

Figure S8. a-d) the SEM images of CoFe@HPC-1000.

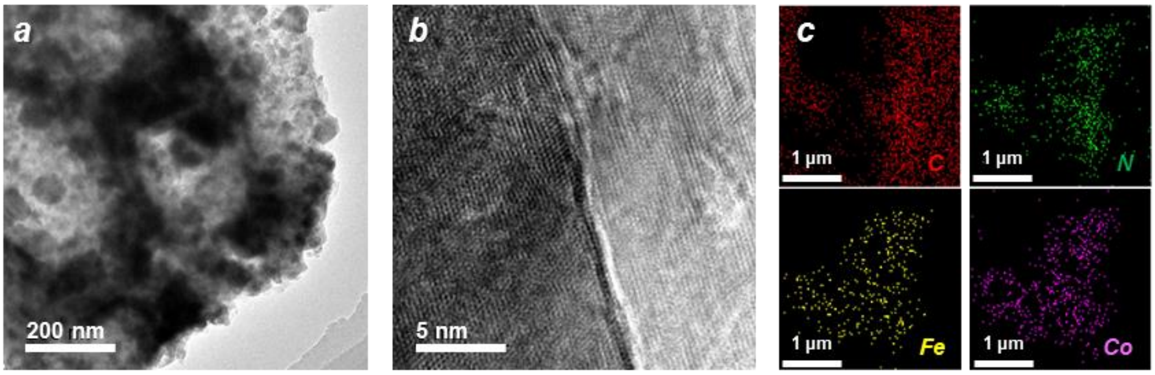

Figure S9. a-b) the TEM images and c) TEM-EDS mapping images of CoFe@HPC-400

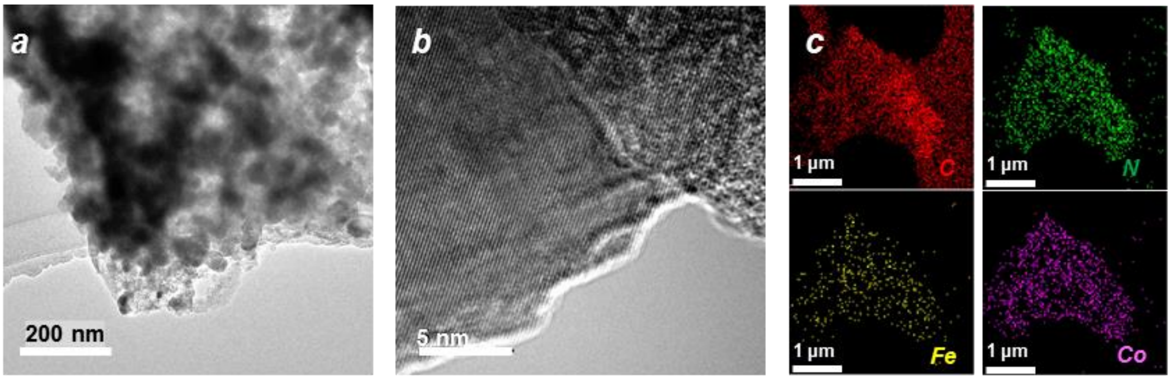

Figure S10. a-b) the TEM images and c) TEM-EDS mapping images of CoFe@HPC-600

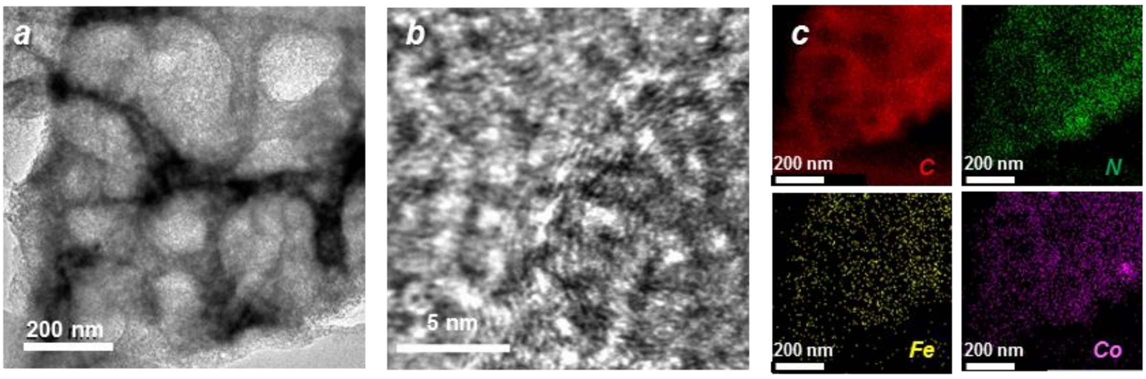

Figure S11. a-b) the TEM images and c) TEM-EDS mapping images of CoFe@HPC-800

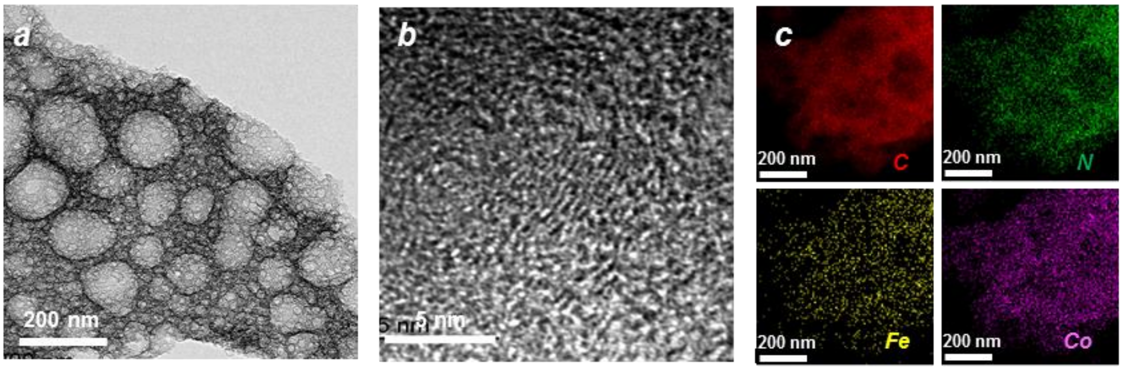

Figure S12. a-b) the TEM images and c) TEM-EDS mapping images of CoFe@HPC-1000

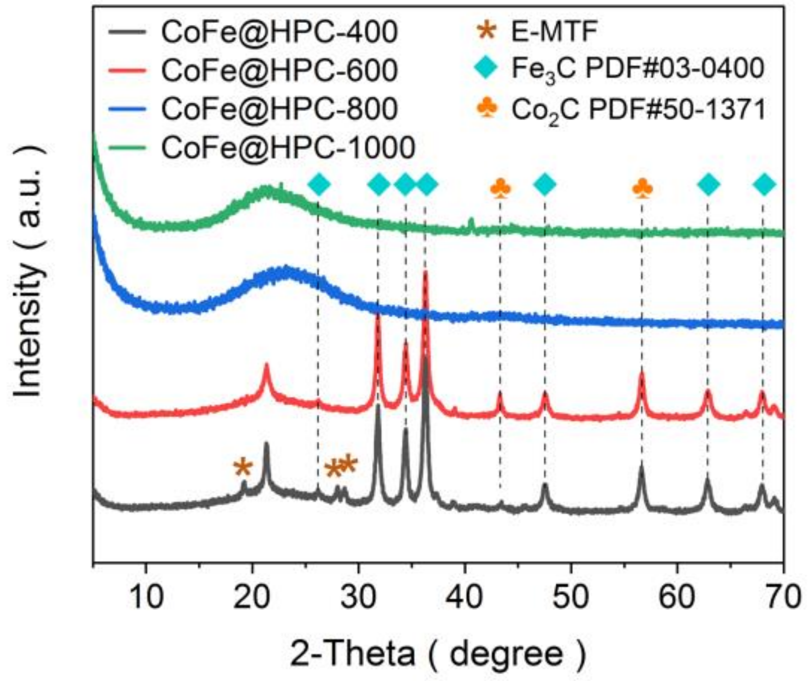

Figure S13. The PXRD results of synthesized CoFe@HPCx.

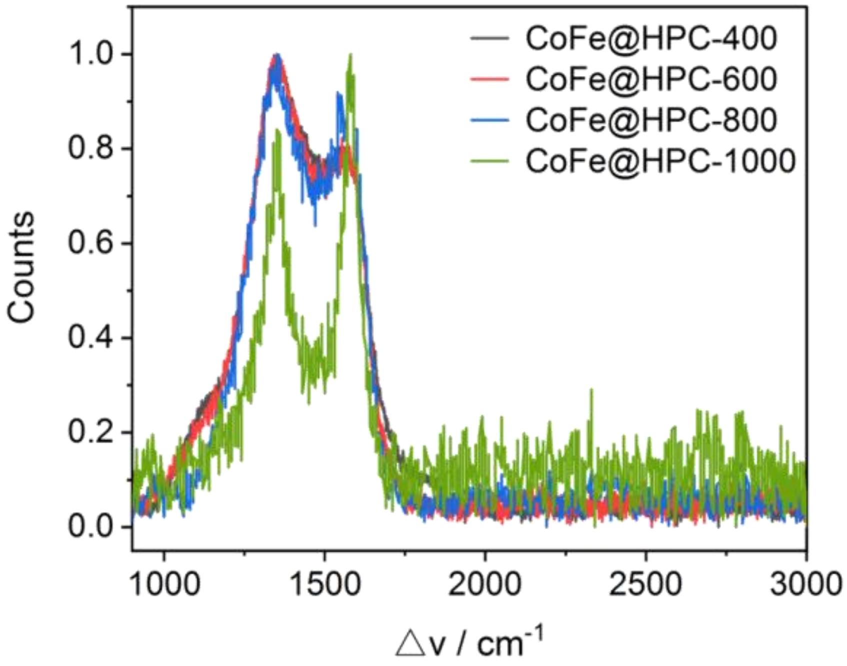

Figure S14. The Raman pattems of CoFe@HPCx.

Table S1. Raman parameters obtained from deconvolution spectra.

Samples	Raman					
	**D1**	**G**	**A_{D3}**	**I_{D1}/I_G**	**R2**	**d_{nc}**
	cm^{-1}	cm^{-1}				nm
CoFe@HPC-400	1341	1568	47.6	1.10	0.51	1.41
CoFe@HPC-600	1340	1567	68.1	1.11	0.51	1.41
CoFe@HPC-800	1340	1535	34.9	0.93	0.40	1.30
CoFe@HPC-1000	1349	1580	80.4	0.82	0.38	1.22

All Raman spectra were fitted with four bands. The frequency of the two bands corresponding to D1 and G were allowed to vary between 1300 and 1400 cm^{-1} and between 1550 and 1650 cm^{-1}, respectively. The frequency of the D3 band for amorphous carbon and D4 band tentatively assigned to polyenes or oligomers were fixed at 1500 cm^{-1} and 1200 cm^{-1}, respectively.

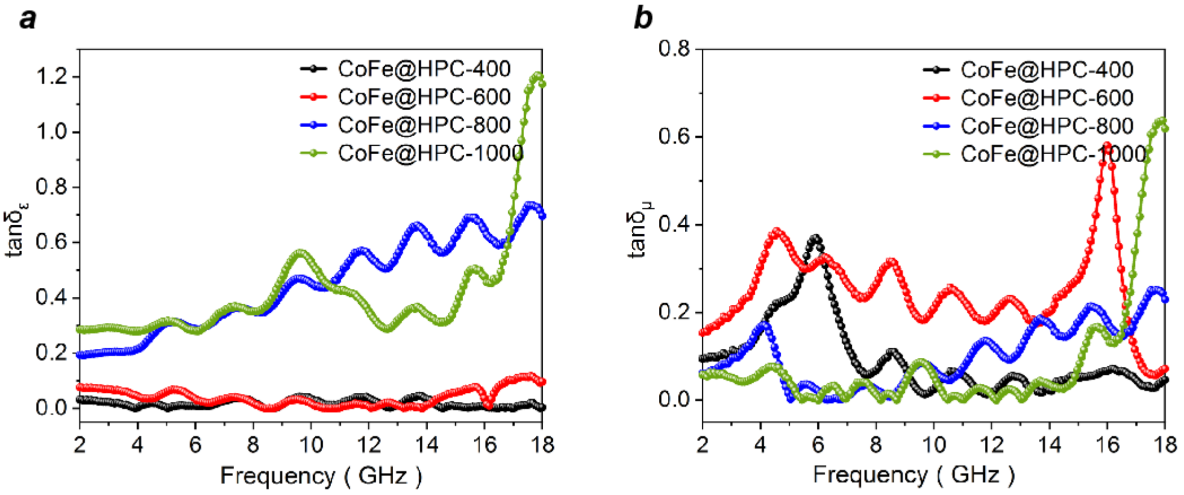

Figure S15. The a) $\tan\delta_\varepsilon$ and b) $\tan\delta_\mu$ values of CoFe@HPCx.

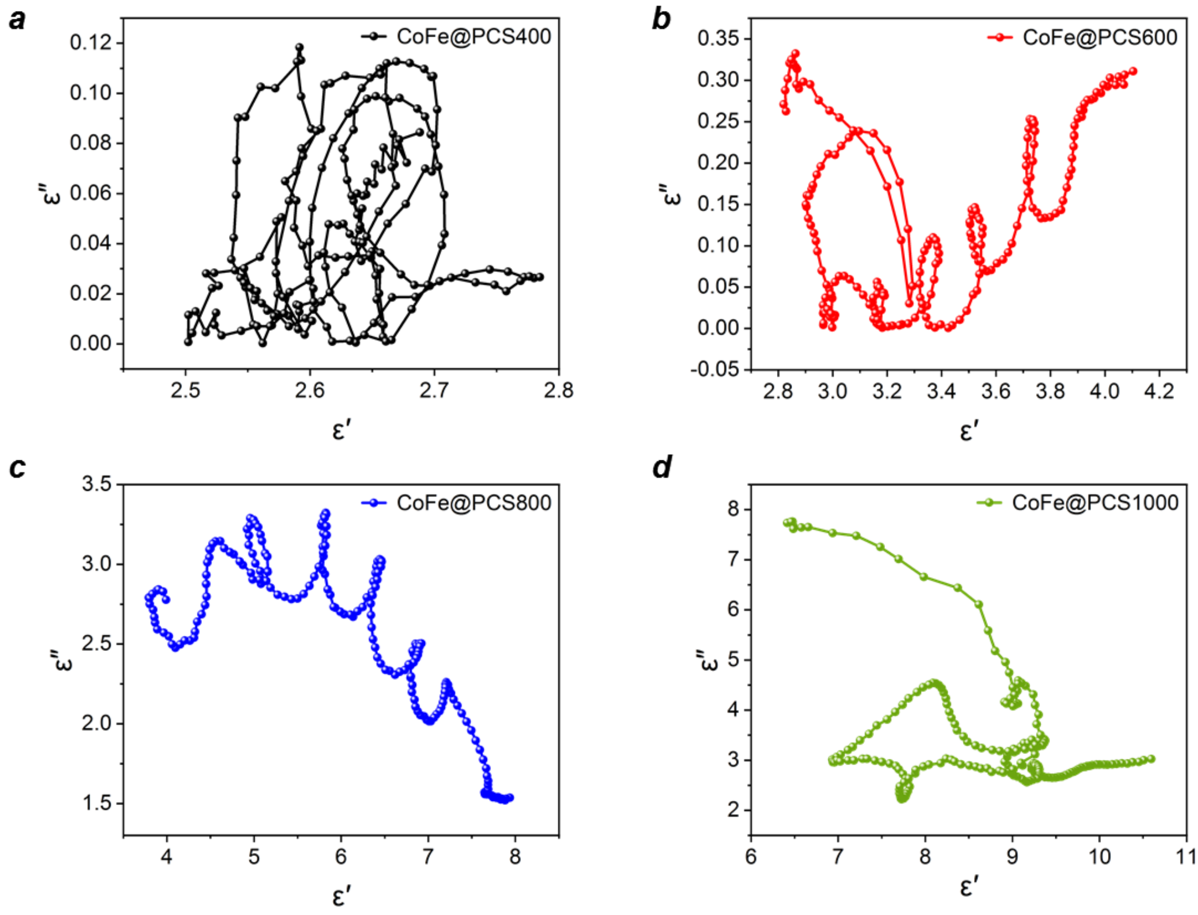

Figure S16. The Cole-Cole curves of CoFe@HPC-400 (a), CoFe@HPC-600 (b), CoFe@HPC-800 (c) and CoFe@HPC-1000 (d).

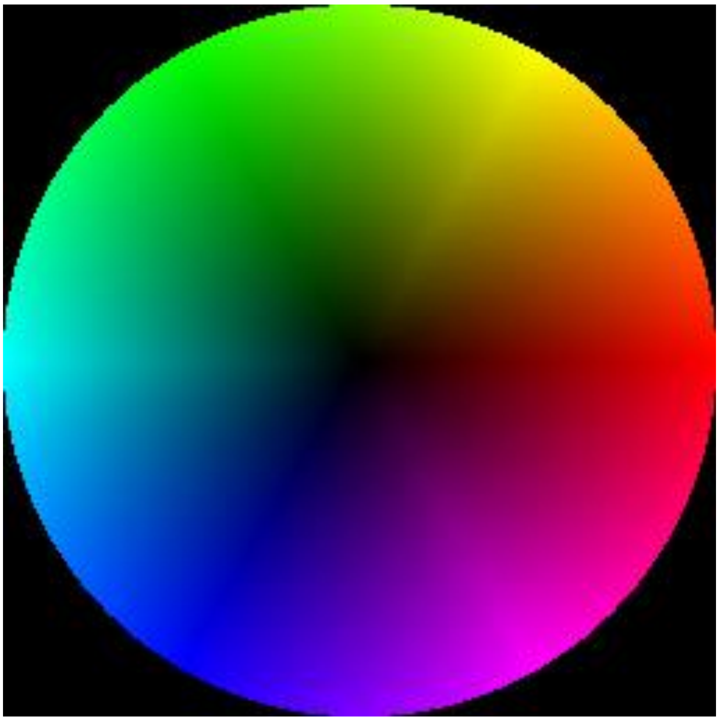

Figure S17. The colour disk for electric field distribution diagram, colour represents direction and brightness represents intensity.

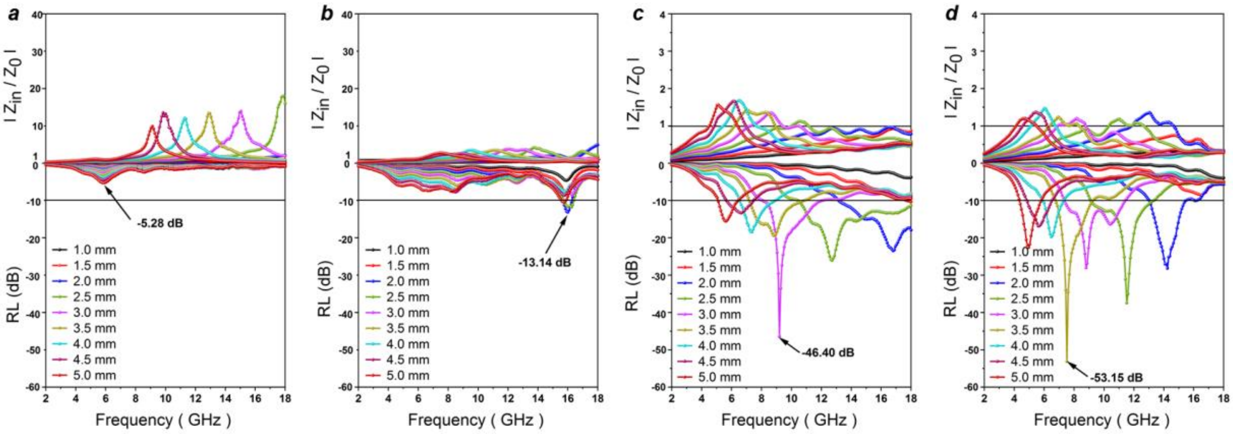

Figure S18. The | Z_{in} / Z_0 | and *RL* plots of CoFe@HPC-400 (a), CoFe@HPC-600 (b), CoFe@HPC-800 (c) and CoFe@HPC-1000 (d).

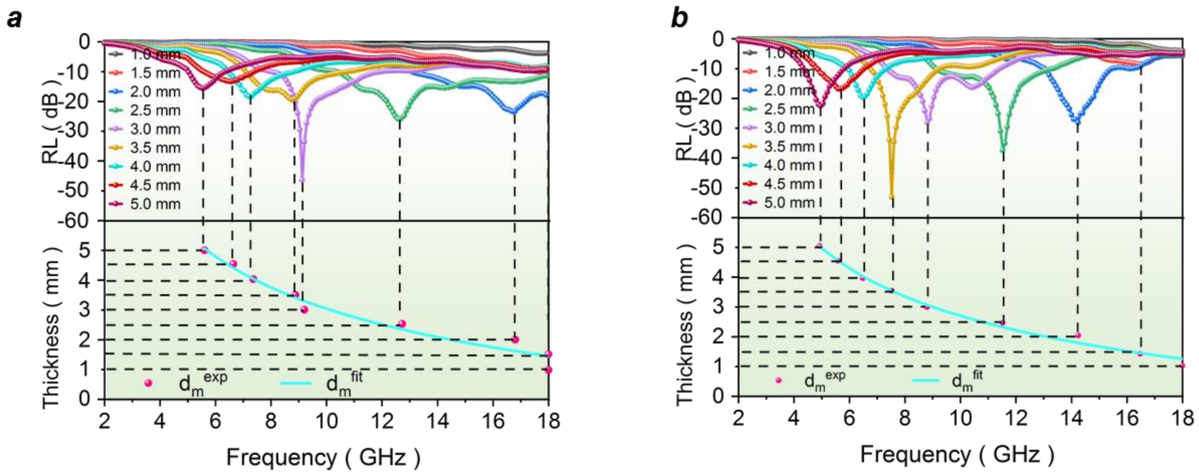

Figure S19. The RL plots and d_m values of CoFe@HPC-800 (a) and CoFe@HPC-1000 (b).

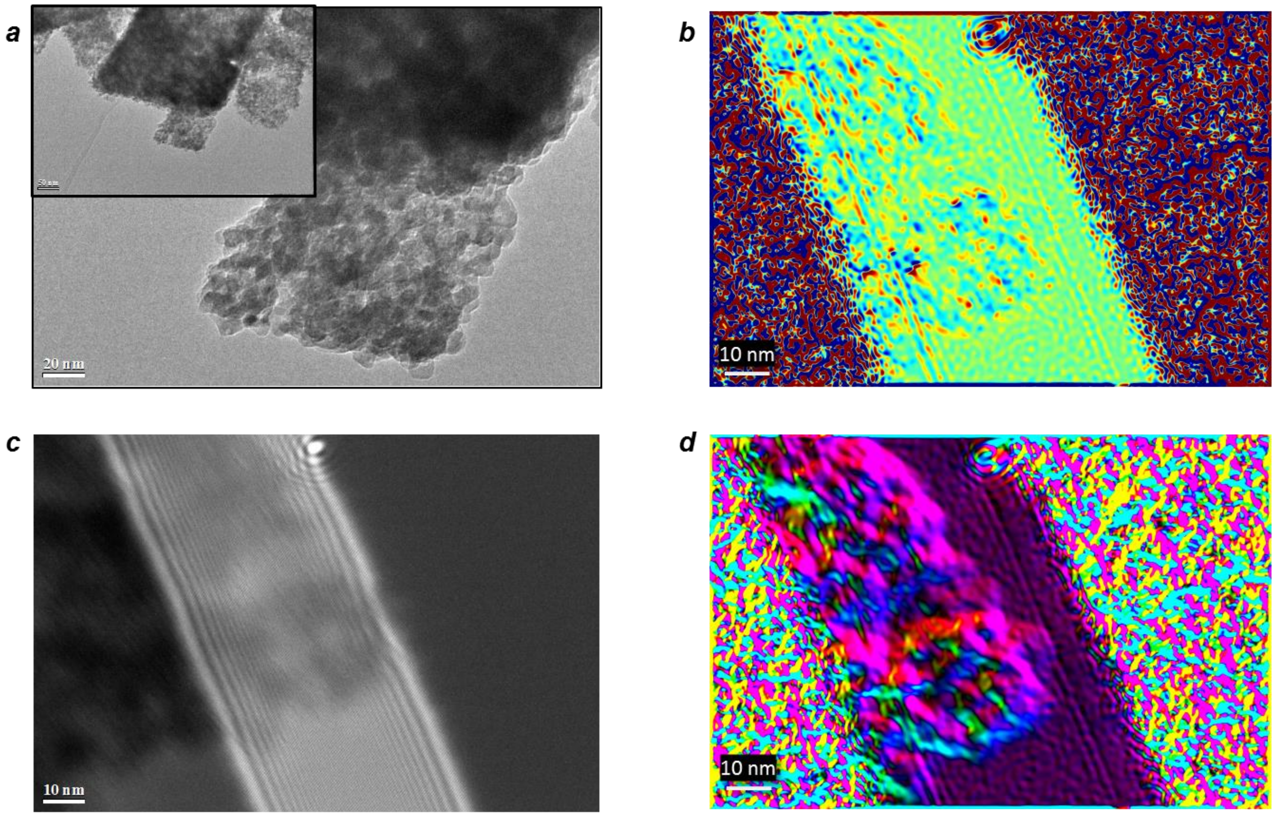

Figure S20. a) The HR-TEM, b) the charge density map, c) hologram image HR-TEM, d) the magnetic domians distribution map of CoFe@HPC-400.

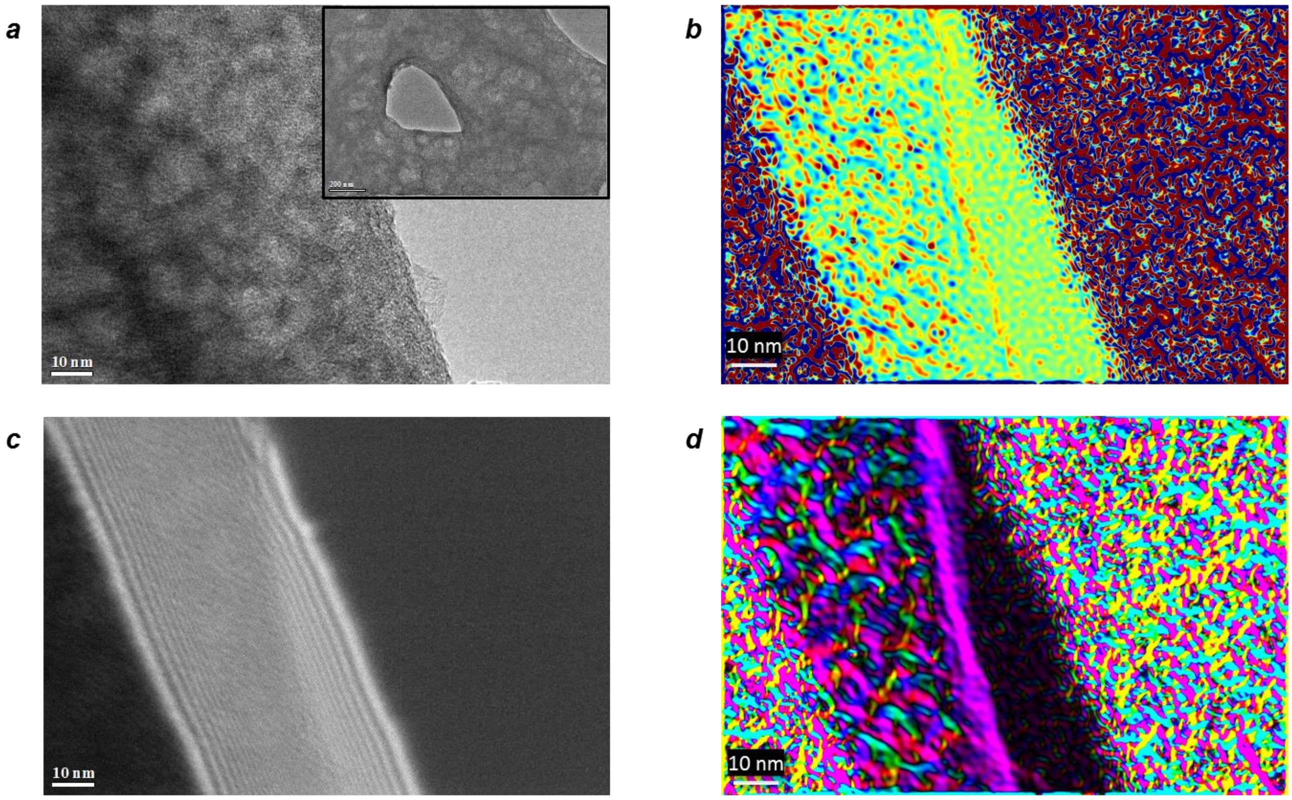

Figure S21. a) The HR-TEM, b) the charge density map, c) hologram image HR-TEM, d) the magnetic domians distribution map of CoFe@HPC-600.

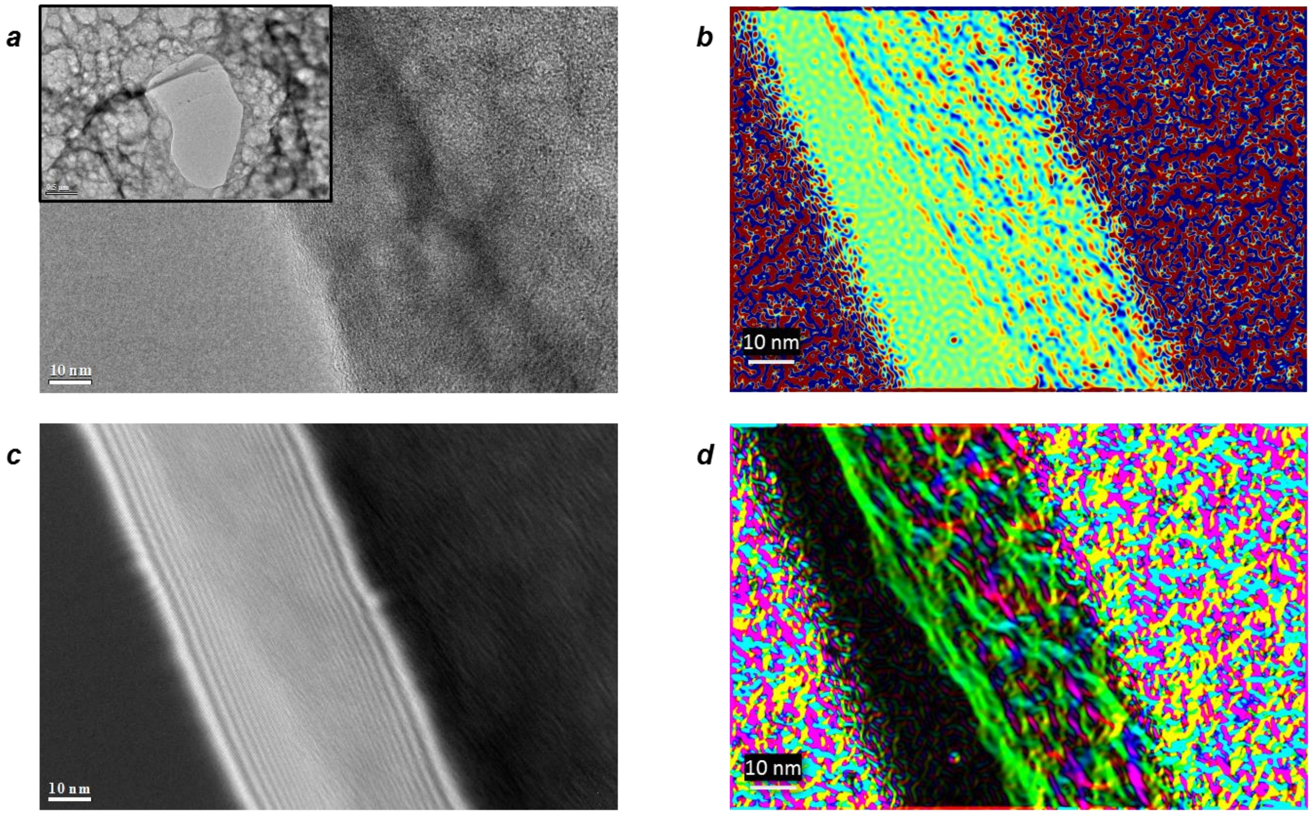

Figure S22. a) The HR-TEM, b) the charge density map, c) hologram image HR-TEM, d) the magnetic domians distribution map of CoFe@HPC-800.

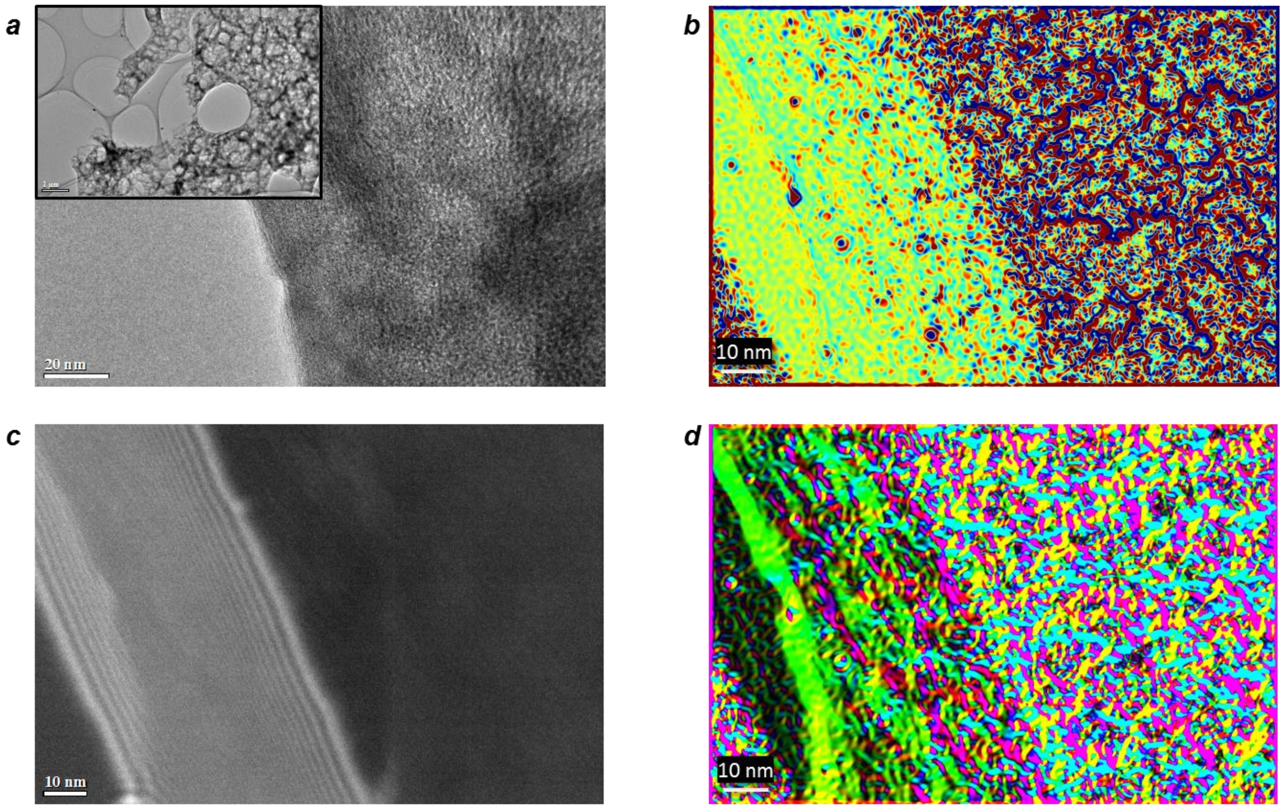

Figure S23. a) The HR-TEM, b) the charge density map, c) hologram image HR-TEM, d) the magnetic domians distribution map of CoFe@HPC-1000.

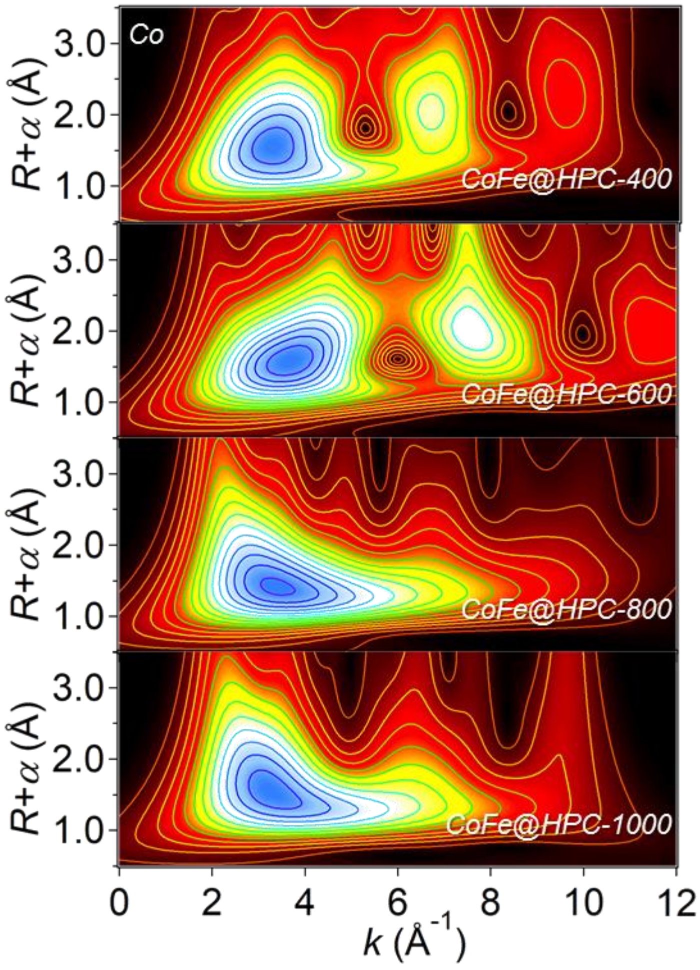

Figure S24. The wavelet transform for the k^2-weighted EXAFS signals of Co atom in CoFe@HPCx.

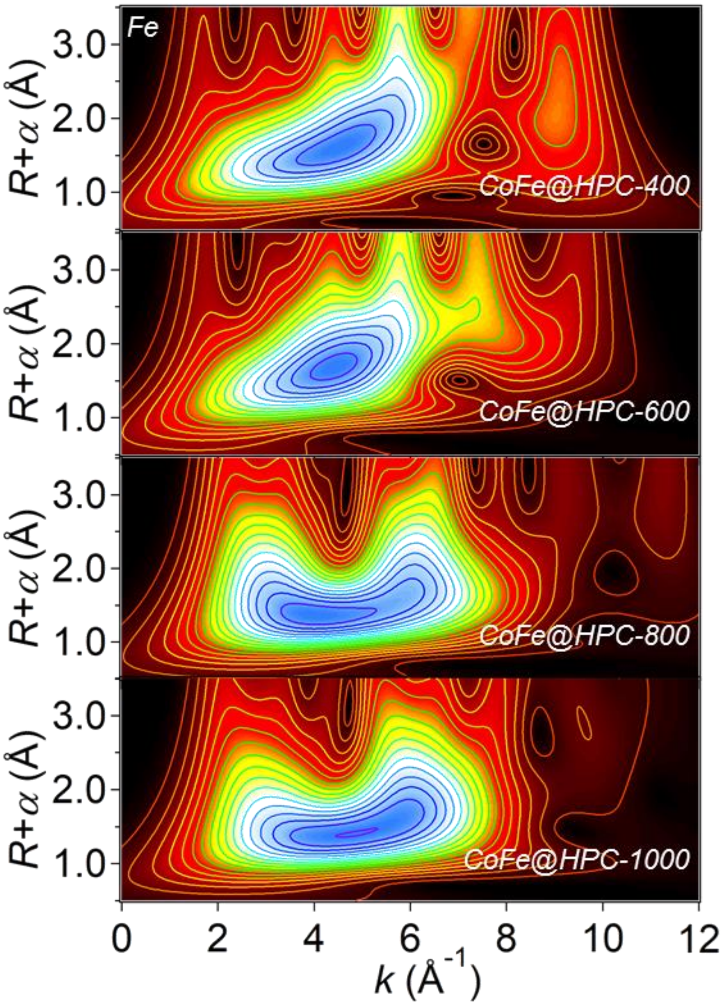

Figure S25. The wavelet transform for the k^2-weighted EXAFS signals of Fe atom in CoFe@HPCx.

Table S2. ICP-OES results of of CoFe@HPCx.

Metal	CoFe@HPC-400 (wt%)	CoFe@HPC-600 (wt%)	CoFe@HPC-800 (wt%)	CoFe@HPC-1000 (wt%)
Co	0.62	0.45	0.36	0.35
Fe	0.56	0.47	0.33	0.32

Table S3. The Fourier transform of K-edge EXAFS parameters of CoFe@HPCx.

Samples	Co			Fe		
	Path	CN	R(Å)	Path	CN	R(Å)
CoFe@HPC-400	Co-N	6.1	1.87	Fe-N	2.5	2.01
	Co-Co	8.2	2.86	Fe-Fe	3.9	2.66
CoFe@HPC-600	Co-N	2.5	1.88	Fe-N	5.3	1.97
	Co-Co	5.4	2.66	Fe-Fe	5.9	2.69
CoFe@HPC-800	Co-N	3.8	1.89	Fe-N	4.2	1.97
	Co-Co	-	-	Fe-Fe	1.3	2.50
CoFe@HPC-1000	Co-N	2.6	1.94	Fe-N	6.6	2.04
	Co-Co	-	-	Fe-Fe	2.6	2.60

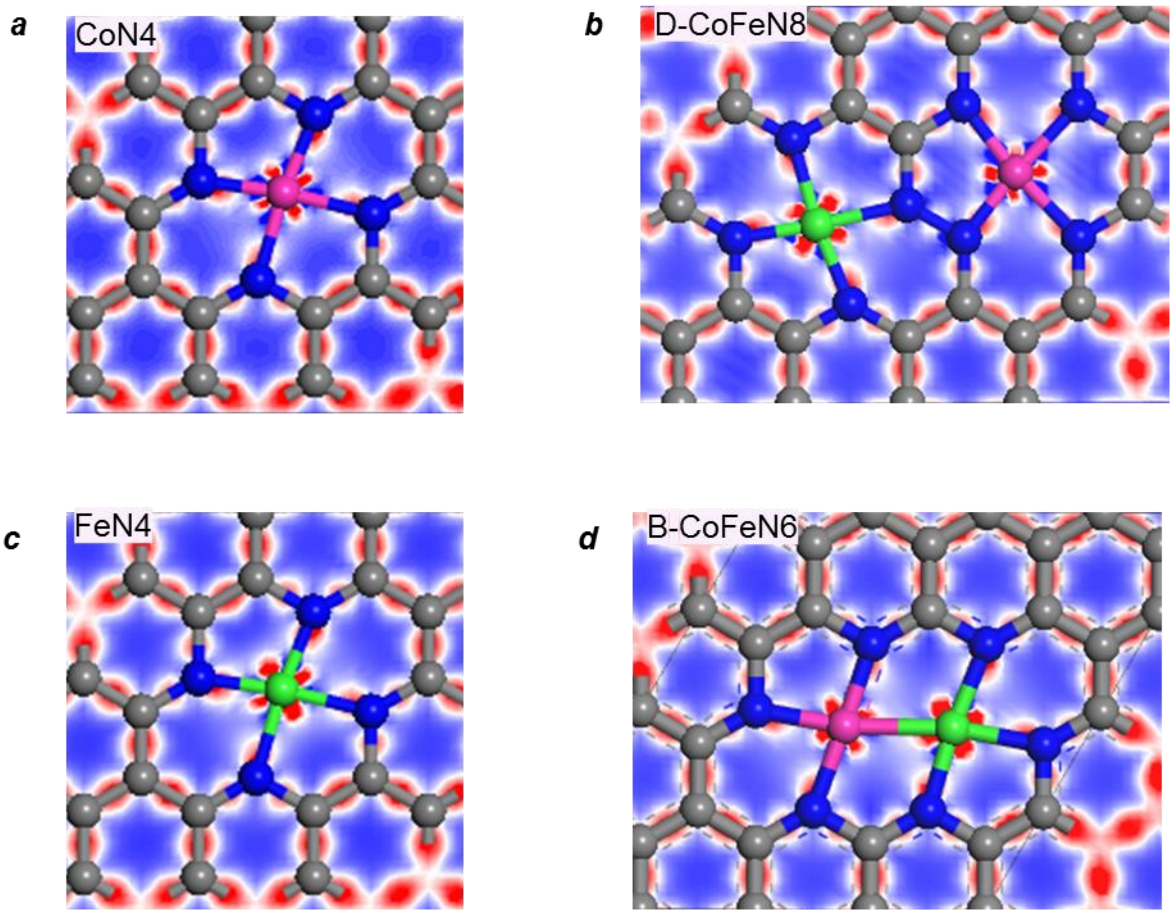

Figure S26. The density functional theory (DFT) theoretical calculations based on the isolated (a) CoN_4, (b) FeN_4, (c) discrete $CoFeN_8$ (D-$CoFeN_8$), and (d) bonding $CoFeN_6$ (B-$CoFeN_6$) models.

Table S4. Comparison of microwave absorption performance of CoFe@HPCx with other MOFs-derived carbon absorbers.

Samples	*RL*	Thickness	Bandwidth	Loading	| *SRL* |	Ref.
	dB	mm	GHz	wt %	dB ·mg^{-1} ·mm^{-1}	
S_m-Fe/Co	-45.20	3.0	5.61	75	20.09	S[1]
Fe/MnO@C	-45.00	4.0	5.00	50	22.50	S[2]
$NiCo_2S_4/Co_{1-x}S/Co(OH)F$	-39.60	4.7	6.03	33.3	25.30	S[3]
FeCo@C	-35.90	4.0	2.58	30	29.92	S[4]
FeCoZn@C NFs-M	-51.20	3.4	2.50	47.5	31.70	S[5]
CoFe@C(PBA)	-44.60	2.15	5.50	60	34.57	S[6]
CuNi-3/ACET	-40.54	2.5	5.87	40	40.54	S[7]
$Fe\&TiO_2@C$	-51.80	3.0	6.50	40	43.17	S[8]
$N-14(NbS_2)$	-43.85	2.5	6.48	40	43.85	S[9]
MnO/Co/C	-68.89	2.64	5.30	50	52.19	S[10]
Ni@C	-59.50	4.5	4.70	25	52.89	S[11]
$Ti_3C_2T_x$/Ni/AS	-59.50	2.0	4.1	50	59.50	S[12]
$CuCo_2S_4/CoS_2/Cu_{1.96}S$	-45.70	2.2	6.80	33.3	62.38	S[13]
$Cu/C@MoS_2$	-48.22	2.5	7.12	30	64.29	S[14]
Fe-Co/NC/rGO	-43.26	2.5	9.12	25	69.22	S[15]
NiCo@C/ZnO	-60.97	2.3	6.08	33.3	79.61	S[16]
Fe@NCNs-11	-64.75	2.7	6.20	30	79.94	S[17]
FeCo/CoFe2O4/C	-52.30	1.95	5.00	30	89.40	S[18]
S0-700($ZnO/Zn_xCo_y/CoO$)	-48.85	1.6	4.80	33.3	91.69	S[19]
CoFe@HPC-1000	-53.15	3.5	4.96	15	101.24	This work
CoFe@HPC-800	-46.40	3.0	7.20	15	103.11	This work

References

[1] Z. Ma, H. Liu, Z. Luo, *Adv. Funct. Mater.* **2023**.

[2] G. He, Y. Duan, H. Pang, *Nano-Micro Letters* **2020**, 12, 57.

[3] J. Liu, L. Zhang, H. Wu, *Adv. Funct. Mater.* **2021**, 31, 2110496.

[4] L. Rao, L. Wang, C. Yang, R. Zhang, J. Zhang, C. Liang, R. Che, *Adv. Funct. Mater.* **2023**.

[5] F. Wu, M. Ling, L. Wan, P. Liu, Y. Wang, Q. Zhang, B. Zhang, *Chem. Eng. J. (Amsterdam, Neth.)* **2022**, 435, 134905.

[6] X. Liang, G. Wang, W. Gu, G. Ji, *Carbon* **2021**, 177, 97.

[7] J. Cheng, H. Zhang, H. Wang, Z. Huang, H. Raza, C. Hou, G. Zheng, D. Zhang, Q. Zheng, R. Che, *Adv. Funct. Mater.* **2022**, 32, 2201129.

[8] B. Deng, Z. Xiang, J. Xiong, Z. Liu, L. Yu, W. Lu, *Nano-Micro Letters* **2020**, 12, 55.

[9] H. Zhang, J. Cheng, H. Wang, Z. Huang, Q. Zheng, G. Zheng, D. Zhang, R. Che, M. Cao, *Adv. Funct. Mater.* **2022**, 32, 2108194.

[10] X. Zhang, X. Tian, C. Liu, J. Qiao, W. Liu, J. Liu, Z. Zeng, *Carbon* **2022**, 194, 257.

[11] L. Wang, M. Huang, X. Yu, W. You, J. Zhang, X. Liu, M. Wang, R. Che, *Nano-Micro Letters* **2020**, 12, 150.

[12] W. Luo, M. Wang, K. Wang, P. Yan, J. Huang, J. Gao, T. Zhao, Q. Ding, P. Qiu, H. Wang, P. Lu, Y. Fan, W. Jiang, *Adv. Sci.* **2022**, 9, 2104163.

[13] J. Liu, L. Zhang, D. Zang, H. Wu, *Adv. Funct. Mater.* **2021**, 31.

[14] Z. Gao, Z. Ma, D. Lan, Z. Zhao, L. Zhang, H. Wu, Y. Hou, *Adv. Funct. Mater.* **2022**, 32, 2112294.

[15] S. Wang, Y. Xu, R. Fu, H. Zhu, Q. Jiao, T. Feng, C. Feng, D. Shi, H. Li, Y. Zhao, *Nano-Micro Letters* **2019**, 11, 76.

[16] J. Wang, Z. Jia, X. Liu, J. Dou, B. Xu, B. Wang, G. Wu, *Nano-Micro Letters* **2021**, 13, 175.

[17] T. Gao, R. Zhao, Y. Li, Z. Zhu, C. Hu, L. Ji, J. Zhang, X. Zhang, *Adv. Funct. Mater.* **2022**, 32, 2204370.

[18] J. Chen, J. Zheng, Q. Huang, F. Wang, G. Ji, *ACS Appl. Mater. Interfaces* **2021**, 13, 36182.

[19] Z. Gao, D. Lan, L. Zhang, H. Wu, *Adv. Funct. Mater.* **2021**, 31.
